# Amyloid‐Forming Amylin Secreted by the Pancreas Promotes Hypoxia‐Inducible Factor Activation and Mitochondrial Alterations in Liver and Heart During Type 2 Diabetes Pathogenesis

**DOI:** 10.1002/cph4.70252

**Published:** 2026-09-04

**Authors:** Alice E. Knapton, Nirmal Verma, Deepak Kotiya, Alice P. Sowton, Benjamin D. Thackray, Lorenz M. W. Holzner, Paula M. Darwin, Sanda Despa, Florin Despa, Andrew J. Murray

**Affiliations:** ^1^ Department of Physiology Development and Neuroscience Cambridge UK; ^2^ Department of Pharmacology and Nutritional Sciences University of Kentucky Lexington Kentucky USA

**Keywords:** diabetes, heart, hypoxia signaling, liver, metabolism, mitochondria

## Abstract

In humans, hypersecretion of amyloidogenic amylin (islet amyloid polypeptide, IAPP) in the setting of insulin resistance and type 2 diabetes (T2D) promotes systemic oligomerization and tissue deposition, with deposits identified in failing human hearts and the cerebrovasculature of patients with Alzheimer's Disease. In the renal vasculature, amylin aggregation disrupts microvascular integrity, activating hypoxia signaling pathways and contributing to maladaptive erythropoietic responses, including excess erythrocytosis. We hypothesized that amyloid‐forming amylin would activate hypoxia‐inducible factor (HIF) signaling, leading to metabolic alterations in liver and heart during T2D pathogenesis. To investigate this, we used the HIP rat, which expresses human amylin specifically in pancreatic β‐cells, comparing tissues from 14 to 16‐month‐old rats with those from age‐matched wild‐type, hyperglycemic UCD and amylin‐knockout (AKO) rats. We found greater accumulation of HIF‐1α and HIF‐2α in HIP rat livers compared with those in other groups, alongside increased expression of HIF‐1 target genes. Mitochondrial ETS capacity was elevated in HIP rat livers, in conjunction with the formation of mitochondrial supercomplexes. Amylin aggregates formed in the hearts of HIP rats, alongside HIF‐1α and HIF‐2α accumulation. This was associated with suppression of ETS capacity, and increased p‐AMPK/AMPK, indicating possible cardiac energetic impairment. Pancreatic secretion of amyloidogenic amylin is thus associated with HIF activation in key metabolic organs beyond the renal vasculature, and occurs alongside mitochondrial alterations in liver and heart that are consistent with sustained hypoxic stress. Our results, alongside previous work, suggest that amylin dysregulation is an overlooked, human‐relevant aspect of diabetes pathogenesis, and as such, a potential therapeutic target.

## Graphical abstract

Human amylin is secreted from the pancreas of HIP rats and is amyloidogenic, forming aggregates systemically. In comparison with age‐matched control (WT), hyperglycemic (UCD) and amylin knockout rats (AKO), HIP rats showed increased hypoxia‐inducible factors (HIF‐1/2) in heart and liver, alongside alterations in mitochondrial electron transfer system (ETS) capacity.

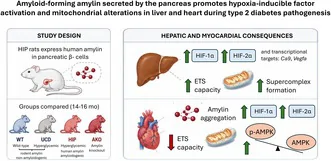

## 1 Introduction

Diabetes‐related dysregulation of amylin (islet amyloid polypeptide, IAPP) represents a critical but often underappreciated human‐relevant pathogenic mechanism that is largely overlooked in conventional animal models of metabolic disease owing to species differences in amylin amyloidogenicity. Importantly, rodent amylin has a very low propensity to form amyloid aggregates, owing to amino acid substitutions in the key amyloidogenic region (Westermark et al. 1990). In humans, however, hypersecretion of amyloidogenic amylin in the setting of insulin resistance and type 2 diabetes (T2D) promotes systemic oligomerization and tissue deposition, extending far beyond the pancreas. As such, dysregulated human amylin has emerged as a systemic amyloidogenic factor linking metabolic disease to multi‐organ microvascular injury and organ dysfunction.

Amylin aggregates have been identified in failing human hearts, where they incorporate into cardiomyocytes and are associated with contractile dysfunction, particularly in individuals with comorbid obesity and heart failure (Despa et al. 2012). In the brain, amylin amyloid deposits accumulate within the cerebrovasculature and parenchyma in association with Alzheimer's dementia (Ly et al. 2021; Verma et al. 2023), implicating circulating metabolic peptides in neurovascular injury and cognitive decline. Similarly, in the kidney, amylin deposition within the renal vasculature disrupts microvascular integrity, thereby activating hypoxia signaling pathways and contributing to maladaptive erythropoietic responses, including excess erythrocytosis (Verma et al. 2020).

This latter finding points towards a novel mechanism of pathogenesis with potentially wider cardiometabolic relevance, whereby amylin‐mediated vascular dysfunction leads to tissue hypoxia and downstream metabolic consequences in key tissues. Tissue hypoxia is a complication common to a great many human diseases (Chen et al. 2020). This includes diseases characterized by impairments in convective oxygen delivery, but importantly many metabolic‐related diseases. For instance, hypoxia is a prominent feature of metabolic dysfunction‐associated steatotic liver disease (MASLD) (Fuster‐Martínez et al. 2026), a highly prevalent condition which is closely associated with obesity and T2D (Younossi et al. 2025). The hypoxia‐inducible factors HIF‐1α and HIF‐2α accumulate in the livers of MASLD patients (Morello et al. 2018; Yu et al. 2020), and influence disease progression via interactions with processes including steatosis, inflammation, and fibrosis (Holzner and Murray 2021). Myocardial hypoxia has also been proposed to play a role in heart failure pathogenesis, with HIF activation playing a protective role in the context of acute myocardial ischemia (Giordano 2005). Impairments in mechanisms of hypoxia sensing and HIF stabilization in the hearts of patients with diabetes may explain the increased susceptibility of these patients to ischemia/reperfusion injury (Heather and Clarke 2011).

Hypoxia, and consequent activation of HIF signaling, has profound effects on cellular metabolism, including increased glycolytic capacity (Semenza et al. 1994), inhibition of pyruvate oxidation (Kim et al. 2006; Papandreou et al. 2006) and suppression of fatty acid oxidation (Cole et al. 2016; Narravula and Colgan 2001). Moreover, a great many HIF‐mediated cellular responses to hypoxia converge on the mitochondrial electron transfer system (ETS), which plays a central role in maintaining cellular energetic and redox function. Of note, metabolic responses to hypoxia are highly context‐dependent, showing both temporal and tissue specificity (Horscroft et al. 2015; Morash et al. 2013; O'Brien et al. 2021).

Here we aimed to investigate the hypothesis that amyloid‐forming amylin secreted from the pancreas activates HIF signaling and alters mitochondrial respiratory function in liver and heart during T2D pathogenesis. To do this, we used the HIP rat, which expresses human amylin specifically in pancreatic β‐cells (Butler et al. 2004), comparing tissues from 14 to 16 month old rats with those from wild‐type (WT) rats, hyperglycemic rats expressing rodent amylin (UCD rats), and amylin‐knockout (AKO) rats, expressing neither human nor rodent forms.

## 2 Materials and Methods

For full methodology, please see Supporting Information.

### 2.1 Animals

Animal work was performed in accordance with the NIH Guide for the Care and Use of Laboratory Animals, with prior approval by the Institutional Animal Care and Use Committee, University of Kentucky.

We compared four groups: HIP rats (*n* = 21), which develop T2D due to expression of amyloid‐forming human amylin (Butler et al. 2004); UCD rats (*n* = 19), which develop obesity, insulin resistance, and diabetes without amyloid (expressing non‐amyloidogenic rat amylin) (Cummings et al. 2008); non‐diabetic wild‐type controls (WT; *n* = 19); and amylin knockout rats (AKO; *n* = 12) (Ly et al. 2017). Rats were studied at 14–16 months of age, and only males were used because HIP females become diabetic at a more advanced age (Butler et al. 2004; Ly et al. 2017).

Rats were single‐housed in individually ventilated cages (ACE, Allentown, NJ, USA) and maintained on a standard chow diet (Teklad Global 18% Protein Rodent Diet #2018, VA, USA) and reverse osmosis drinking water for the duration of the study. All animals were maintained on a 12:12 h light: dark cycle at temperatures between 21°C and 24°C.

Rats were culled by a single terminal surgical excision of the heart whilst under deep isoflurane anesthesia (3%–5% isoflurane in 100% oxygen). Adequate depth of anesthesia was verified by a lack of reflex upon toe pinch. Organs were collected using sterile surgical instruments and embedded in paraffin or snap‐frozen in liquid nitrogen before being stored at −80°C.

**FIGURE 1:**
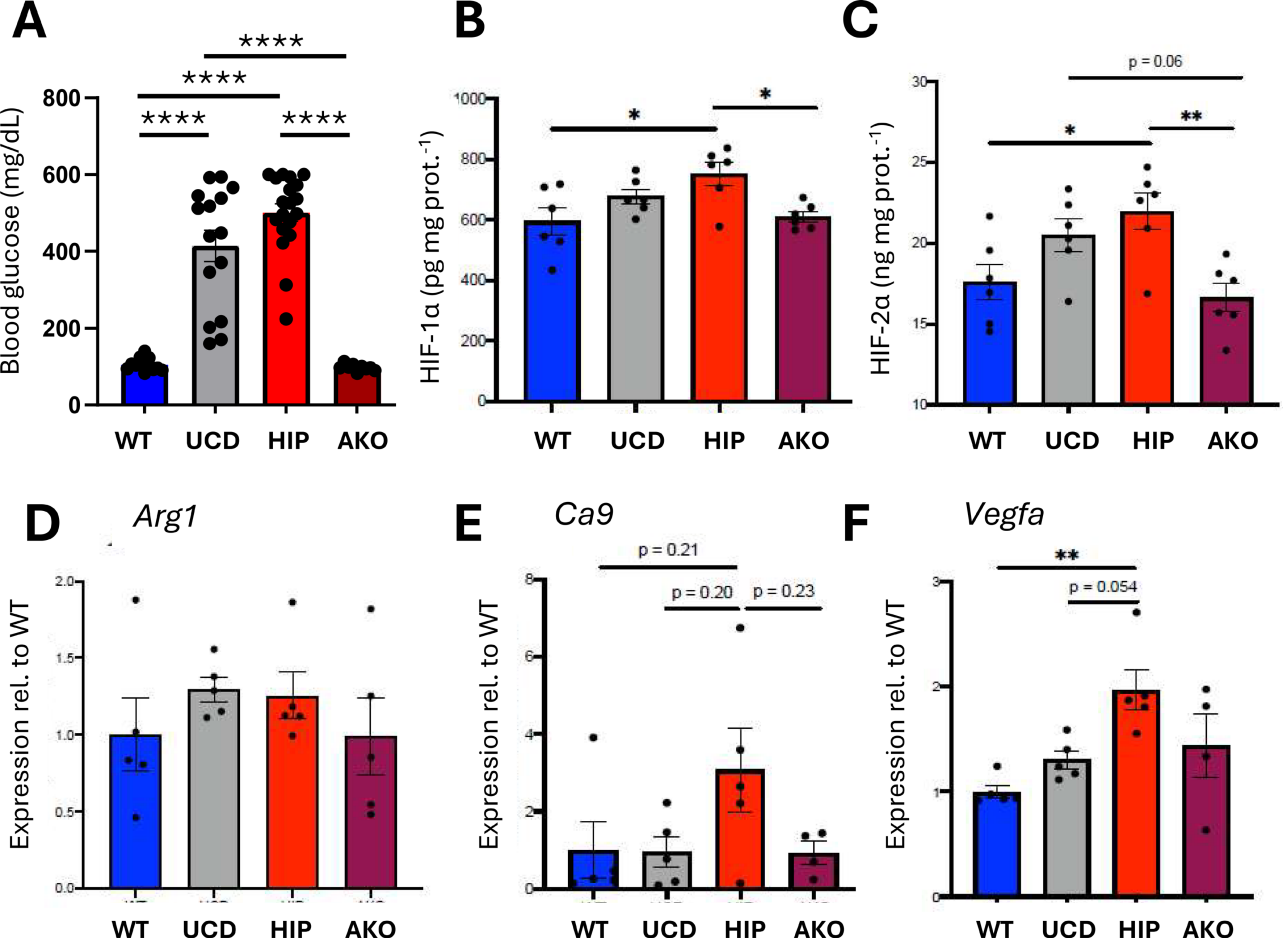
Hypoxia Inducible Factor (HIF) accumulation in the livers of human amylin‐expressing HIP rats. (A) Blood glucose concentrations in wild‐type, hyperglycemic (UCD), human amylin‐expressing (HIP), and amylin‐knockout (AKO) rats. Concentrations of (B) HIF‐1α and (C) HIF‐2α measured by ELISA in the livers of WT, UCD, HIP and AKO rats (*n* = 6 per group). Expression (relative to wild‐type rats) of HIF target genes (D) *Arg1* (encoding arginase 1), (E) *Ca9* (encoding carbonic anhydrase 9) and (F) *Vegfa* (encoding vascular endothelial growth factor a), (*n* = 4–5 per group). One‐way ANOVA with post hoc Tukey's multiple comparison test. Data represented as mean ± SEM. * *p* < 0.05, **p < 0.01, ***p < 0.001.

Rats in the four groups were matched by age (WT—16.4 ± 0.3 months, HIP—15.1 ± 0.4 months, UCD—14.5 ± 0.3 months, and AKO—15.1 ± 1.7 months). The diabetic HIP and UCD rats were also matched by blood glucose levels at the time of euthanasia (505 ± 23 and 415 ± 42 mg/dL, respectively), with these levels being higher than those in WT rats (105.4 ± 5 mg/dL) or AKO rats (97.7 ± 3 mg/dL; Figure 1A).

### 2.2 Respirometry

Respirometry was performed on homogenates prepared from previously‐frozen liver and heart to assess electron transfer system (ETS) capacity, using a published protocol, previously validated in livers and hearts from normoxic and hypoxic rats (Knapton et al. 2026).

Respiration supported by the N‐pathway via complex I was stimulated with NADH (1.25 mM for liver, 4.4 mM for heart; N‐linked), followed by succinate to additionally stimulate the S‐pathway via complex II (10 mM for liver, 12.5 mM for heart; NS‐linked). Complex I was inhibited by rotenone (2.5 μM for liver, 0.5 μM for heart), allowing assessment of S‐pathway dependent ETS capacity (complex II only; S‐linked). Antimycin A (5 μM) was added to inhibit complex III, isolating electron flux through complex IV which was assessed using ascorbate (2 mM) and TMPD (0.5 mM), with rates corrected for auto‐oxidation by subsequent addition of sodium azide (100 mM).

### 2.3 Immunohistochemistry

Paraffin‐embedded heart blocks (WT, UCD and HIP rats) were cut into 5 μm thick sections. Tissue sections were deparaffinized in xylene and rehydrated two times in serial dilutions of alcohol to 1× PBS. Sections were incubated in 1× retrieval buffer (S1699; Dako), blocked in 15% horse serum, and incubated overnight with an anti‐amylin primary antibody (SC‐377530, Santa Cruz; 1:200 dilution). After washing, sections were incubated with biotinylated goat anti‐mouse IgG secondary antibody (BA2000, Vector lab; 1:400 dilution), followed by HRP conjugated avidin‐biotin complex (ABC kit; PK‐6100; Vectastain laboratories, CA) and then developed in 3‐amino‐9‐ethylcarbazole (AEC; K3461; Dako; CA) chromogen according to the manufacturer's protocol. Sections were again blocked in 10% normal goat serum and incubated overnight with an anti‐HIF‐2α antibody (ab109616, abcam; 1:100 dilution), followed by an AP conjugated anti‐rabbit IgG secondary antibody (A3687, Sigma; 1:50 dilution). Then, sections were developed in StayGreen/AP Plus (ab156428; abcam) chromogen according to manufacturer's protocol. Finally, sections were mounted in aqueous mounting media and imaged with a Nikon Eclipse 55i upright microscope.

### 2.4 Immunofluorescence

Heart sections were prepared as described in the Immunohistochemistry section. Sections were then blocked in 10% goat serum, 5% BSA, and 0.5% Triton X‐100 and washed in PBS. Sections were incubated with an anti‐human amylin antibody (T‐4157, Bachem‐Peninsula Laboratories; 1:200 dilution) overnight at 4°C, then washed three times for 5 min with 0.1% Triton in Tris‐NaCl. Sections were incubated with Alexa Fluor 488 conjugated anti‐rabbit IgG secondary antibody (A‐11008; Invitrogen; 1:500 dilution) for 1 h. The sections were again washed three times for 5 min with 0.1% Triton in Tris‐NaCl, and autofluorescence was blocked in 0.1% Sudan black (BP109‐10, Fisher) for 10 min and washed in 1× PBS thrice. They were cover‐slipped in mounting media with DAPI (ab 104139, Abcam; MA) and imaged with a confocal microscope.

### 2.5 HIF‐1α and HIF‐2α ELISAs

For the detection of HIF‐1α and HIF‐2α in tissue homogenates, Rat Hypoxia Inducible Factor 1 Alpha (HIF‐1α) or Rat Hypoxia Inducible Factor 2 Alpha (HIF‐2α) ELISA kits (MyBioSource) were used according to manufacturer's instructions. The plate was read at 450 and 630 nm within 10 min using a FLUOstar Omega plate reader (BMG Labtech).

### 2.6 Amylin ELISA

A competitive ELISA assay kit (EIA‐AMY‐1, Ray‐biotech) was used to measure the amylin content in whole heart homogenates from age‐matched male WT, HIP, and UCD rats. The protocol followed the manufacturer's instructions.

### 2.7 Enzyme Activity Analysis

Citrate synthase activity was assayed in tissue homogenates as previously described (Houle‐Leroy et al. 2000). Homogenate was prepared in citrate synthase assay buffer (20 mM Tris‐Base, 0.1 mM 5,5‐dithiobis‐2‐nitrobenzoic acid (DTNB), pH 8.0). Acetyl CoA (0.6 mM) was added, and the mixture warmed at 37°C for 3 min, before baseline absorbance was read on a spectrophotometer at 412 nm for 3 min. Oxaloacetate (0.5 mM) was then added to initiate the reaction:

Acetyl CoASH+Oxaloacetate+H2O→CoASH+Citrate

This is coupled to the following reaction:

CoASH+DTNB→TNB+CoSSTNB

Thionitrobenzoic acid (TNB) absorbance was recorded at 412 nm. Baseline rate was subtracted from the reaction rate to calculate the enzyme‐dependent rate of absorbance change, and enzyme activity was calculated as described previously (Horscroft et al. 2015).

### 2.8 Immunoblotting

Frozen tissue was disrupted and homogenized in lysis buffer (20 mM Tris–HCl pH 7.5, 1% Triton X‐100, 1 mM EGTA, 150 mM NaCl, 2.5 mM Na_4_O_7_P_2_.10H_2_O, 1 mM β‐glycerophosphate, 10 mM Na_3_VO_4_, protease inhibitor tablets). Protein concentration was then determined using the Pierce BCA Protein Assay kit (Thermo Scientific). From this, samples were diluted in lysis buffer to give 15 μL aliquots containing 25 μg protein. LDS Sample Buffer (4×, pre‐diluted 1:2 in dH_2_O) and β‐mercaptoethanol, at 2.5% of total volume, were added before 15 μL was loaded into 4%–12% Bis Tris gels in the XCell SureLock Mini‐Cell Electrophoresis System. Proteins were separated by running for 2.5 h at 100 V. Proteins were transferred onto PVDF membranes using a TE77 PWR Semi‐Dry Transfer Unit. Ponceau staining was performed to enable total protein quantification in each sample lane.

#### 2.8.1 OXPHOS Immunoblotting

The membrane was blocked for 1 h at room temperature in 5% BSA, TBS‐Tween (TBS‐T) before incubation with the Total OXPHOS primary antibody for 1 h at room temperature (MitoProfile Total OXPHOS Blue Native Antibody Cocktail, Abcam, Cambridge, UK; 1:1000 in 5% BSA in TBS‐T). After washing with 3 rounds of TBS‐T for 5 min each, the membrane was incubated with an appropriate secondary antibody for 30 min at room temperature (Rb anti‐ms IgG Secondary Antibody HRP conjugate, Invitrogen; 1:20,000 in TBS‐T). A further three rounds of washing with TBS‐T, each for 5 min, took place. Detection involved incubation with chemiluminescent horseradish peroxidase substrate (Pierce ECL Western Blotting Substrate, Life Technologies, Paisley, UK) for 5 min before imaging using Amersham Hyperfilm ECL film (Cytiva) and X‐ray developer Fujifilm FPN‐100.

#### 2.8.2 AMPK/p‐AMPK Western Blotting

The membrane was blocked for 1 h at room temperature in 5% BSA, TBS‐T, before incubation with p‐AMPK primary antibody overnight at 4°C (p‐AMPKalpha, Cell Signaling, Technology, 1:1000 in 5% BSA in TBS‐T + 0.02% Azide + 50 mM NaF). After washing with 3 rounds of TBS‐T for 5 min each, the membrane was incubated with a secondary antibody for 30 min (Rb anti‐ms IgG Secondary Antibody HRP conjugate, Invitrogen; 1:20,000 in TBS‐T). A further three rounds of washing with TBS‐T, each for 5 min, took place. Detection involved incubation with chemiluminescent horseradish peroxidase substrate (Pierce ECL Western Blotting Substrate, Life Technologies, Paisley, UK) for 5 min before imaging using Amersham Hyperfilm ECL film (Cytiva) and X‐ray developer Fujifilm FPN‐100. Following this the membrane was placed in stripping buffer (15 g Glycine, 1 g SDS, 1% Tween‐20, pH 2.2, into 1 L dH_2_O) for 2 × 10 min. The membrane was re‐probed for total AMPK primary antibody overnight at 4°C (AMPKalpha, Cell Signaling Technology, 1:1000 in 5% BSA in TBS‐T + 0.02% Azide), and detection carried out as above.

### 2.9 Bn‐Page

This protocol for isolating and staining for mitochondrial supercomplexes was based on a previous protocol using small gels (Jha et al. 2016).

Snap‐frozen liver (18–25 mg) was prepared as described (Jha et al. 2016). Protein concentration was determined using the Pierce BCA Protein Assay kit (Thermo Scientific). Samples were diluted in isolation buffer to give aliquots containing 50 μg protein. BN‐PAGE was carried out using 3%–12% NativePAGE Bis‐Tris, Mini Protein gels (NuPAGE, Novex, Invitrogen) with the addition of NativeMark Unstained Protein Standard (Invitrogen). Analysis was performed using gels stained with Colloidal Blue. Band identification was achieved through immunoblotting.

### 2.10 Protein Carbonylation

Carbonyl content was determined in heart samples using a Protein Carbonyl Content Assay Kit (Abcam) according to the manufacturer's instructions.

### 2.11 RT‐PCR

RNA was extracted from 30 mg frozen cardiac and liver samples using the RNeasy fibrous tissue mini kit (Qiagen, Manchester, UK), as per the manufacturer's instructions. RNA was then quantified using a Nanodrop 2000 spectrophotometer (Thermo Fisher Scientific). RNA was reverse transcribed to cDNA using the QuantiTect Reverse Transcription kit (Qiagen), as per the manufacturer's instructions. All incubations were performed using the QuantStudio 1 Real‐Time PCR System (Applied Biosystems, Thermo Fisher Scientific).

Real time qPCR was conducted using the QuantiNova SYBR Green PCR kit (Qiagen) and QuantiTect Primer Assays (Qiagen). The details of specific primers are given in Table S1. Each individual sample, pooled sample serial dilution step, and control was plated in triplicate, with the corresponding SYBR Green Master Mix and primer for each gene on a 384‐well plate. qPCR was then carried out using a LightCyclerR 480 System (Roche). Genes of interest (*Vegfa*, *Ca9*, *Arg1*, *Higd1a*) were normalized to the geometric mean of three reference genes (*18S*, *Actb*, *Yhwaz*) and the fold change in gene expression was calculated using the relative standard curve method from the pooled samples.

### 2.12 Statistics

Results were analyzed using a one‐way analysis of variance (ANOVA) test to determine significant differences between experimental groups, unless otherwise stated. This was followed by a Tukey's post hoc test to compare individual group pairs. Where data was not normally distributed, for example, analysis of amylin deposits by immunofluorescence, a Kruskal–Wallis test was used, followed by Dunn's post hoc test to compare individual group pairs. Differences were considered significant when *p* < 0.05.

## 3 Results

### 3.1 Amyloid‐Forming Amylin Is Associated With Hepatic HIF Activation

We initially sought to understand whether amyloid‐forming amylin, secreted by the pancreas, promoted hepatic hypoxia signaling. HIF‐1α and HIF‐2α protein levels were measured in hepatic homogenates using ELISA. Hepatic HIF‐1α was 26.1% higher in HIP rats compared with WT rats (*p* < 0.05), and 22.9% higher than in AKO rats (*p* < 0.05; Figure 1B). Hepatic HIF‐2α was 24.9% higher in HIP rats compared with WT rats (*p* < 0.05) and 32.1% higher compared with AKOs (*p* < 0.01; Figure 1C). There was no difference in HIF‐1α or HIF‐2α levels between UCD rat livers and those of any other group, although there was a non‐significant trend towards higher levels of HIF‐2α in comparison with AKO rat livers (*p* < 0.06).

To understand whether HIF activation also resulted in upregulation of HIF target genes, hepatic expression of *Arg1* (encoding arginase 1), *Ca9* (encoding carbonic anhydrase 9), and *Vegfa* (encoding vascular endothelial growth factor a) were measured. Expression of *Arg1* was not different between groups (Figure 1D). *Ca9* showed a non‐significant trend towards greater expression in HIP rat livers compared to UCD rat livers (*p* = 0.20; Figure 1E). Expression of *Vegfa* was 97.3% greater in HIP rat livers compared with WT rat livers (*p* < 0.01), with a trend towards greater expression compared with UCD rat livers (*p* = 0.054; Figure 1F).

Taken together, these results indicated that amyloid‐forming amylin expressed in the pancreas of HIP rats was associated with hepatic activation of HIF‐1 and HIF‐2, with increased expression of the HIF‐1 target gene *Vegfa*.

### 3.2 Amyloid‐Forming Amylin Increased ETS Capacity and Mitochondrial Supercomplexes in Liver

**FIGURE 2:**
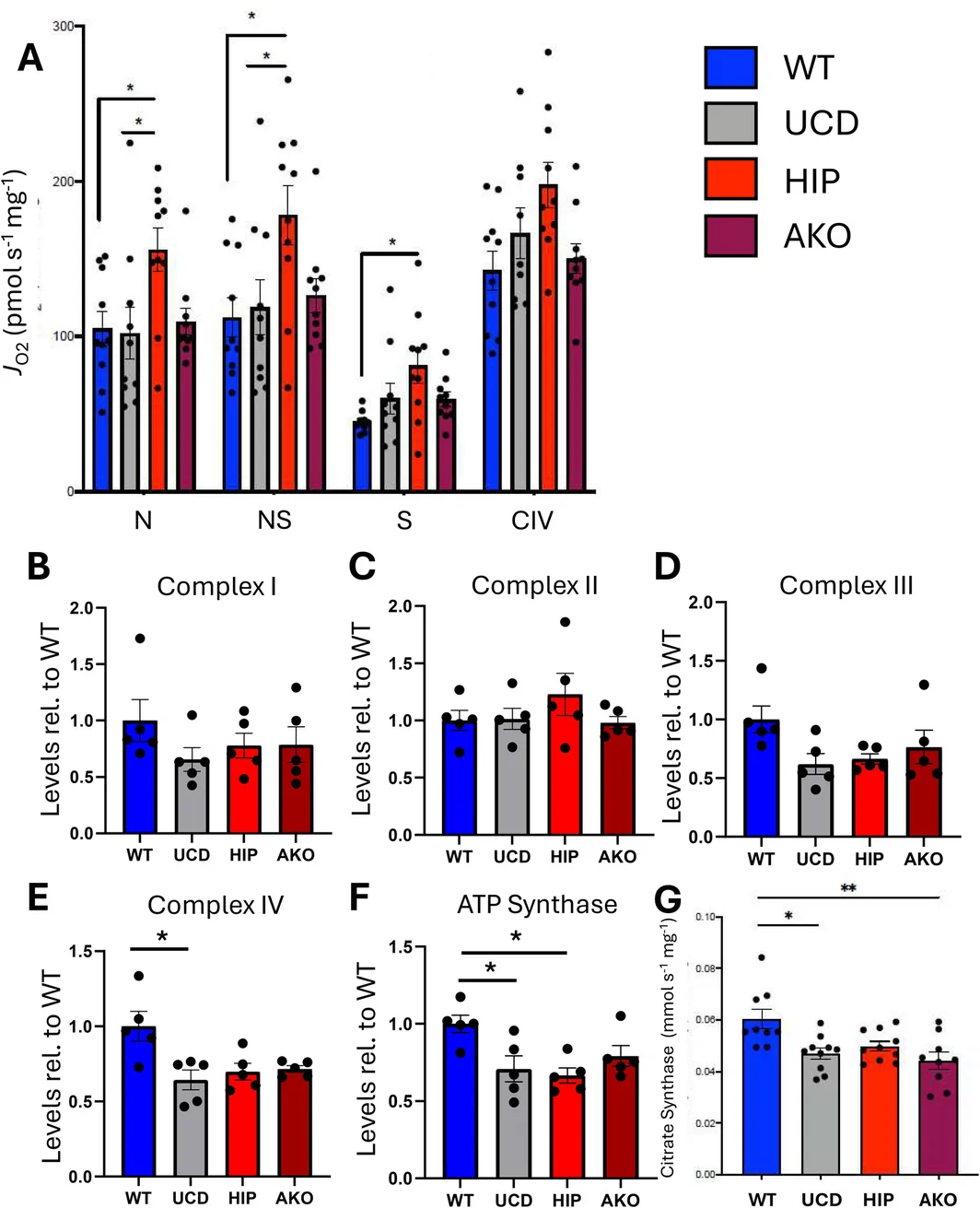
Hepatic electron transfer system (ETS) activity, mitochondrial protein levels and citrate synthase activity. (A) Oxygen flux (*J*_O2_) in liver homogenates from wild‐type (WT), hyperglycemic (UCD), human amylin‐expressing (HIP) and amylin‐knockout (AKO) rats. Fluxes measured in the presence of NADH (supporting complex I; N), NADH and succinate (supporting complex I and II; NS), succinate with rotenone (supporting complex II; S) and TMPD with ascorbate (supporting complex IV) (*n* = 10 per group). Hepatic levels of representative subunits of (B) complex I (NDUFA9), (C) complex II (SDHA), (D) complex III (UQRC2), (E) complex IV (MTCO1), (F) ATP synthase (ATP5A), all expressed relative to WT (*n* = 5 per group). (G) Hepatic citrate synthase activities (*n* = 9–10 per group). One‐way ANOVA with post hoc Tukey's multiple comparison test. Data represented as mean ± SEM. *p < 0.05, **p < 0.01.

Next, we aimed to understand whether hepatic HIF activation in HIP rats was associated with alterations in mitochondrial ETS capacity. Respiratory capacity was measured in homogenates of rat liver using high‐resolution respirometry and an approach optimized for use with frozen tissue homogenates (Knapton et al. 2026). In HIP rat liver homogenates, respiratory capacities of the N‐pathway via complex I (supported by NADH), the NS‐pathway via complexes I&II (supported by NADH and succinate) and the S‐pathway via complex II (supported by succinate in the presence of rotenone) were 48.4% (*p* < 0.05), 58.8% (*p* < 0.05), and 80.1% (*p* < 0.05) higher, respectively, than in homogenates of WT rat livers (Figure 2A). Further, respiration through the N‐pathway and the NS‐pathway were 52.9% (*p* < 0.05) and 50.2% (*p* < 0.05) higher, respectively, in HIP rat livers than those of UCD rats (Figure 2A). There were no significant differences between groups in the capacity for complex IV respiration (supported by ascorbate and TMPD and corrected for autoxidation using sodium azide). Finally, there were no significant differences in respiratory capacities for any state between WTs, UCDs, and AKOs (Figure 2A).

We aimed to understand whether differences in hepatic mitochondrial content or ETS protein complexes could explain the altered respiratory capacity of HIP rats compared with other rats. There was no difference in the protein levels of representative subunits of ETS complexes I‐IV (NDUFA9, SDHA, UQRC2, MTCO1) between HIP rat livers and those of any other group (Figure 2B–E). Levels of ATP5A (a subunit of ATP‐synthase) were 31.3% lower in HIP rats compared with WT rats (*p* < 0.05), and also 26.9% lower in UCD rats than in WT rats (*p* < 0.05; Figure 2F). UCD rats also had 32.6% (*p* < 0.05) lower levels of MTCO1 compared to WTs (Figure 2E).

We measured citrate synthase activity in hepatic homogenates as a marker of mitochondrial content and found no difference between HIP rats and other groups (Figure 2G). However, citrate synthase activity was 28.1% lower in UCD rats than in WT rats (*p* < 0.05), and 36.4% lower in AKO rats compared with WT rats (*p* < 0.01). To understand whether the observed differences in ETS capacity in HIP rats could be accounted for by differences in mitochondrial content, respiration rates for all rats were expressed relative to hepatic citrate synthase activity from the same rat (Figure S1). Intrinsic respiratory capacity supported by the S‐pathway (corrected to citrate synthase activity) was higher in HIP rats compared to WT rats activity (*p* < 0.05), whilst there was a trend towards increased intrinsic respiration via the NS‐pathway (*p* = 0.06).

These results therefore suggested that the enhanced respiratory capacity in HIP rats compared with WT rats was at least in part due to intrinsic remodeling of the mitochondrial ETS. Previous work from our group found that shorter‐term hypoxia was associated with enhanced hepatic respiratory capacity in association with the formation of mitochondrial supercomplexes (O'Brien et al. 2021). These structures are formed from the higher‐level association of ETS complexes in varying stoichiometries. They include the respirasome, which comprises complex I, a dimer of complex III and complex IV (SCI_1_III_2_IV_1_) (Gu et al. 2016; Letts et al. 2016), but also other structures featuring subunit combinations that can exclude complex I, for example, SCIII_2_IV_1_ (Vercellino and Sazanov 2021).

**FIGURE 3:**
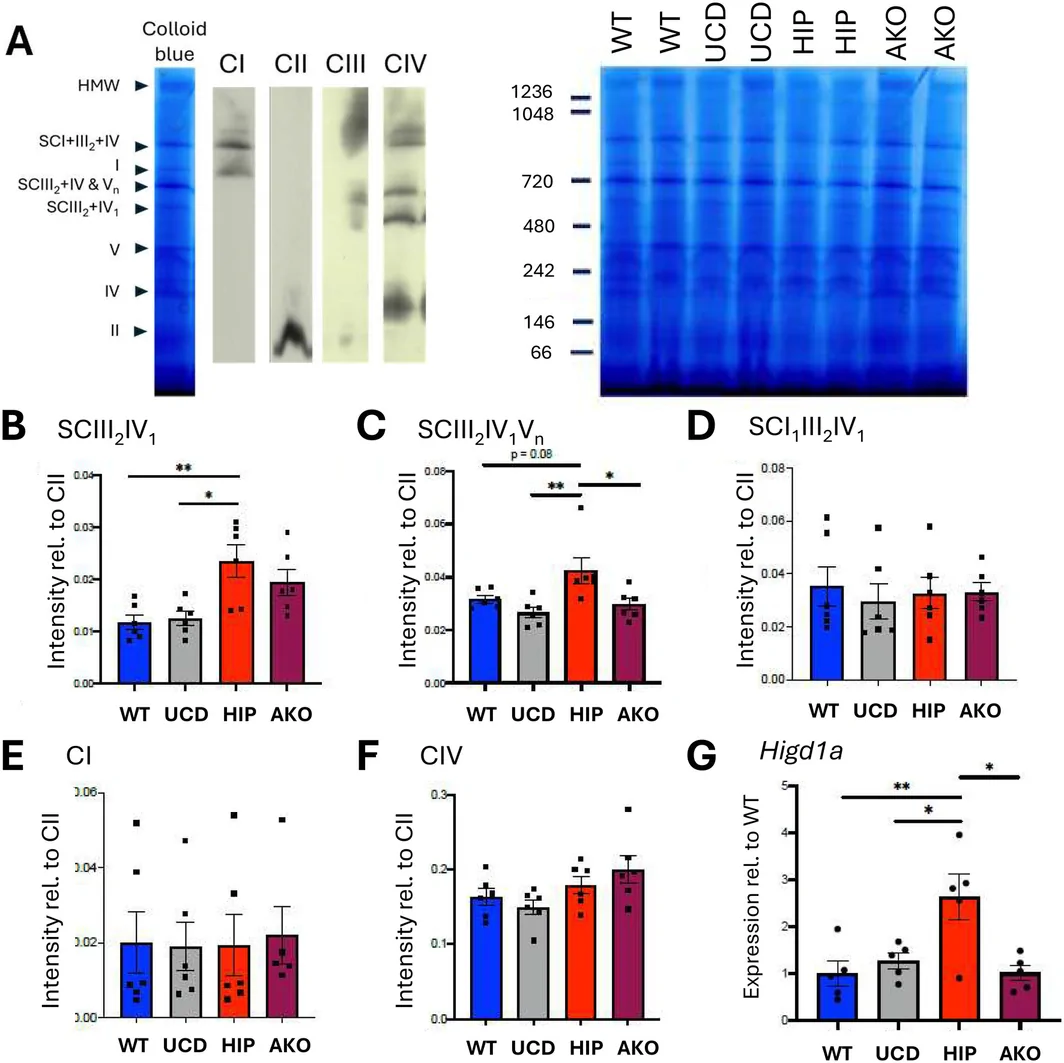
Mitochondrial supercomplex levels and assembly in rat livers. (A) Colloidal blue staining of representative BN‐PAGE gel, and immunoblotting to confirm band identity. Quantification of band intensity (corrected to complex II) for: (B) SCIII_2_IV_1_, (C) SCIII_2_IV_1_V_n_, (D) SCI_1_III_2_IV_1_, (E) complex I, and (F) complex IV in mitochondria isolated from livers of wild‐type (WT), hyperglycemic (UCD), human amylin‐expressing (HIP), and amylin‐knockout (AKO) rats, (*n* = 6 per group). (G) Expression of *Higd1a* (relative to WT), (*n* = 5 per group). One‐way ANOVA with post hoc Tukey's multiple comparison test. Data represented as mean ± SEM. *p < 0.05, **p < 0.01.

We therefore measured ETS supercomplex levels in digitonin‐treated mitochondrial extracts from rat liver homogenates using BN‐PAGE (Figure 3A). In HIP rat livers, the abundance of SCIII_2_IV_1_ was 98.9% greater than in WT rat livers (*p* < 0.01), and 86.3% greater than in UCD rat livers (*p* < 0.05; Figure 3B). Further, hepatic abundance of SCIII_2_IV_1_V_n_ in HIP rats was 59.2% greater than in UCD rats (*p* < 0.01) and 43.1% greater than in AKOs (*p* < 0.05), with a non‐significant trend towards being 33.8% higher than in WT rats (*p* = 0.08; Figure 3C). The abundance of SCI_1_III_2_IV_1_ (Figure 3D) and complexes I and IV in isolation (Figure 3E,F) were not different between groups.

The assembly and stability of large supercomplexes is dependent on mitochondrial cristae shape (Cogliati et al. 2013) regulated through oligomerization of the inner membrane optic atrophy 1 (OPA1) (Patten et al. 2014) in association with hypoxia‐induced gene domain protein‐1a (HIGD1A) (An et al. 2013). Hepatic expression of *Higd1a* was 2.6‐fold higher in HIP rats than in WT rats (*p* < 0.01) and AKO rats (*p* < 0.05), and 2‐fold higher than in UCD rats (*p* < 0.05; Figure 3G).

Our findings therefore revealed that HIF activation in the livers of HIP rats, provoked by amyloid‐forming amylin, was associated with enhanced formation of mitochondrial ETS supercomplexes and enhanced respiratory capacity, consistent with previous findings in rats exposed to shorter‐term hypoxia (2 days, 10% O_2_) (O'Brien et al. 2021).

### 3.3 Amylin Deposition and HIF Activation in Hearts From HIP Rats

Amylin deposition has been identified in the vasculature and interstitial space of failing human hearts. Here we sought to investigate whether amylin accumulates in the hearts of HIP rats, and whether this provokes HIF activation.

**FIGURE 4:**
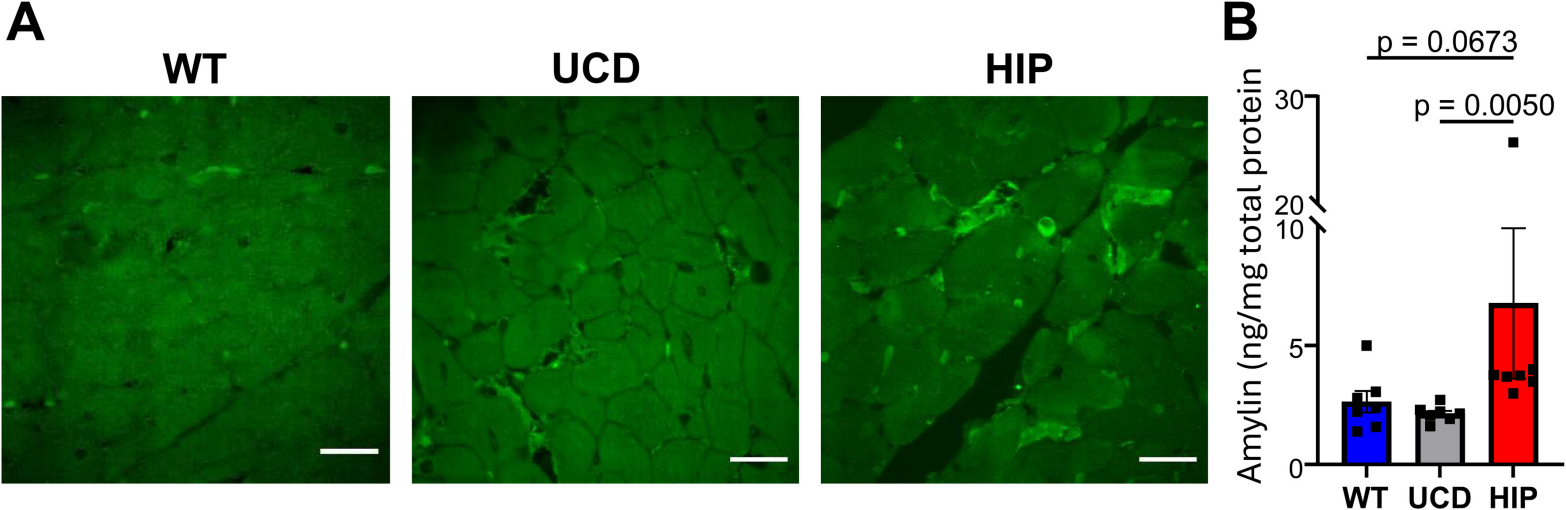
Amylin accumulation in the hearts of HIP rats. (A) Representative immunofluorescence images of heart sections from wild‐type (WT), hyperglycemic (UCD), human amylin‐expressing (HIP) rats stained with an anti‐amylin antibody. Scale bar = 20 μm. (B) Amylin levels, measured by ELISA, in heart homogenates from age‐matched WT, UCD and HIP rats. Statistical analysis used the Kruskal–Wallis test (*p* = 0.0019) followed by Dunn's post hoc test to compare individual group pairs (*n* = 7 per group). Data represented as mean ± SEM.

**FIGURE 5:**
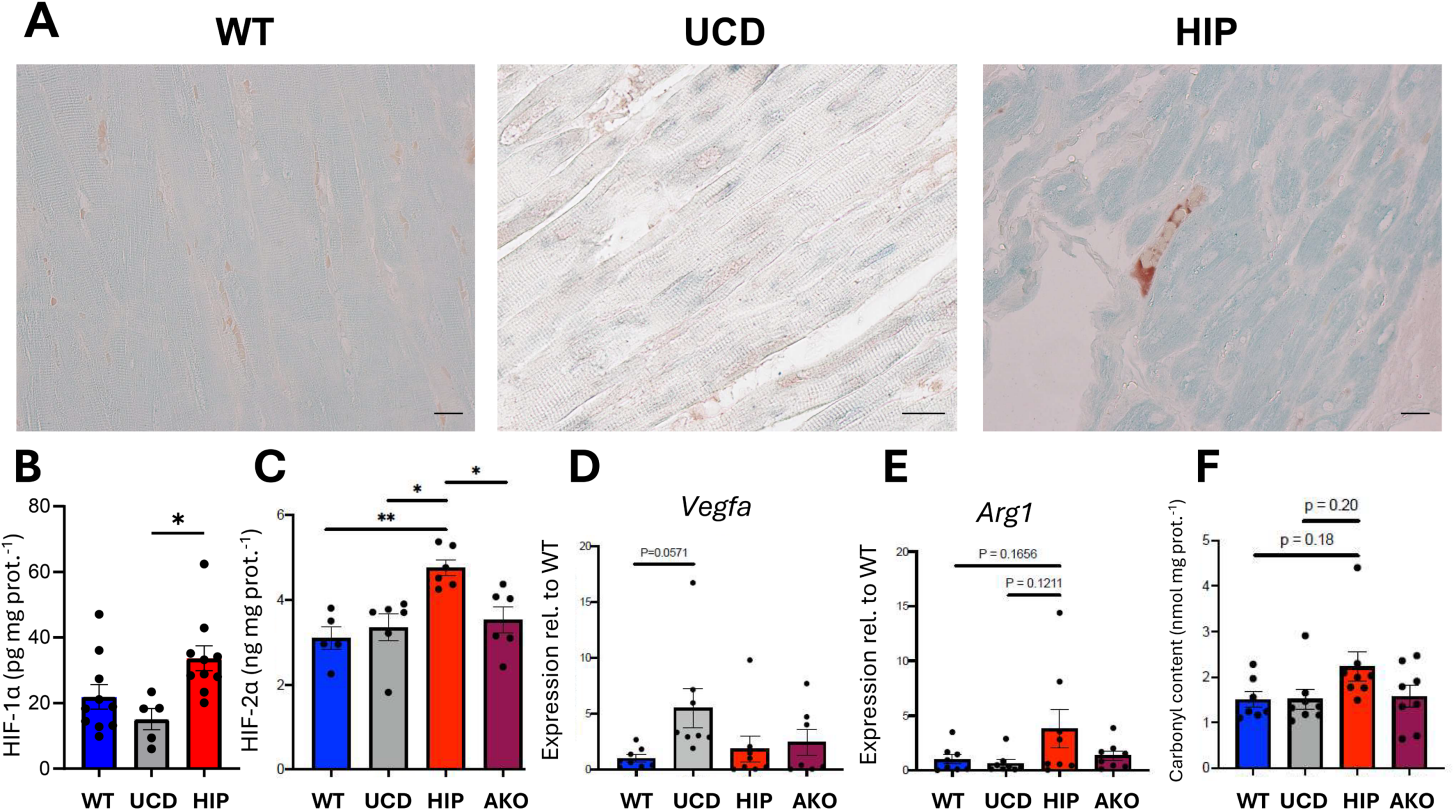
Hypoxia Inducible Factor (HIF) accumulation in the hearts of human amylin‐expressing HIP rats. (A) Representative immunohistochemistry images of heart sections from wild‐type (WT), hyperglycemic (UCD), human amylin‐expressing (HIP) rats dual‐stained with antibodies against amylin (red/brown signal) and HIF‐2α (green signal). Scale bar—20 μm. (B) HIF‐1α measured by ELISA in the hearts of WT, UCD, and HIP rats and (C) HIF‐2α in the hearts of WT, UCD, HIP, and amylin‐knockout (AKO) rats (*n* = 5–10 per group). Expression (relative to wild‐type rats) of HIF target genes (D) *Vegfa* (encoding vascular endothelial growth factor a), (E) *Arg1* (encoding arginase 1), (*n* = 7–8 per group). (F) Protein carbonyl levels in heart homogenates, measured using immunoblotting (*n* = 6 per group). One‐way ANOVA with post hoc Tukey's multiple comparison test. Data represented as mean ± SEM. *p < 0.05, **p < 0.01.

Immunofluorescence revealed significant amylin deposits in the hearts of 14–16 month old HIP rats, which were not present in age‐matched WT counterparts or age‐ and blood‐glucose level matched UCD rats (Figure 4A). In whole heart homogenates, ELISA measurements revealed significantly greater amylin abundance in HIP rats than WT and UCD counterparts (*p* < 0.01; Figure 4B). Immunohistochemistry staining suggested that HIF‐2α levels were higher in hearts from HIP rats compared to WT and UCD rats (Figure 5A). ELISA measurements on heart homogenates revealed that HIF‐1α protein levels were 2‐fold higher in HIP rats compared with UCDs (*p* < 0.05) (Figure 5B), whilst HIF‐2α protein levels were 58.2% higher in HIP rats compared with WTs (*p* < 0.01), 41.8% higher than in UCD rats (*p* < 0.05), and 35.0% higher than in AKO rats (*p* < 0.05; Figure 5C).

To understand the transcriptional implications of HIF activation, expression of the canonical HIF‐1α target *Vegfa* and HIF‐2α target *Arg1* was investigated in the left ventricle. Expression of *Vegfa* showed a non‐significant trend towards being higher in UCD rat hearts compared to WTs (*p* = 0.0571), but there was no difference in expression in HIP rats compared with any other group (Figure 5D). There was a trend towards increased *Arg1* expression in HIP rat hearts compared with WTs (*p* = 0.1656) and UCDs (*p* = 0.1211; Figure 5E). Myocardial protein carbonylation was quantified as a marker of oxidative stress, and although this did not reveal significant differences between any groups, there was a non‐significant trend towards increased protein carbonyl levels in the hearts of HIP rats compared with WT rats (*p* = 0.18) and UCD rats (*p* = 0.20) (Figure 5F).

### 3.4 Amyloid‐Forming Amylin Is Associated With Mitochondrial and Energetic Alterations in HIP Rat Hearts

The heart is highly dependent on mitochondrial oxidative phosphorylation to meet the energetic demands of contractile function, and we therefore used high‐resolution respirometry and a protocol optimized for frozen tissue homogenates (Knapton et al. 2026) to understand whether amylin deposits and HIF stabilization were associated with altered mitochondrial ETS function in HIP rat hearts.

**FIGURE 6:**
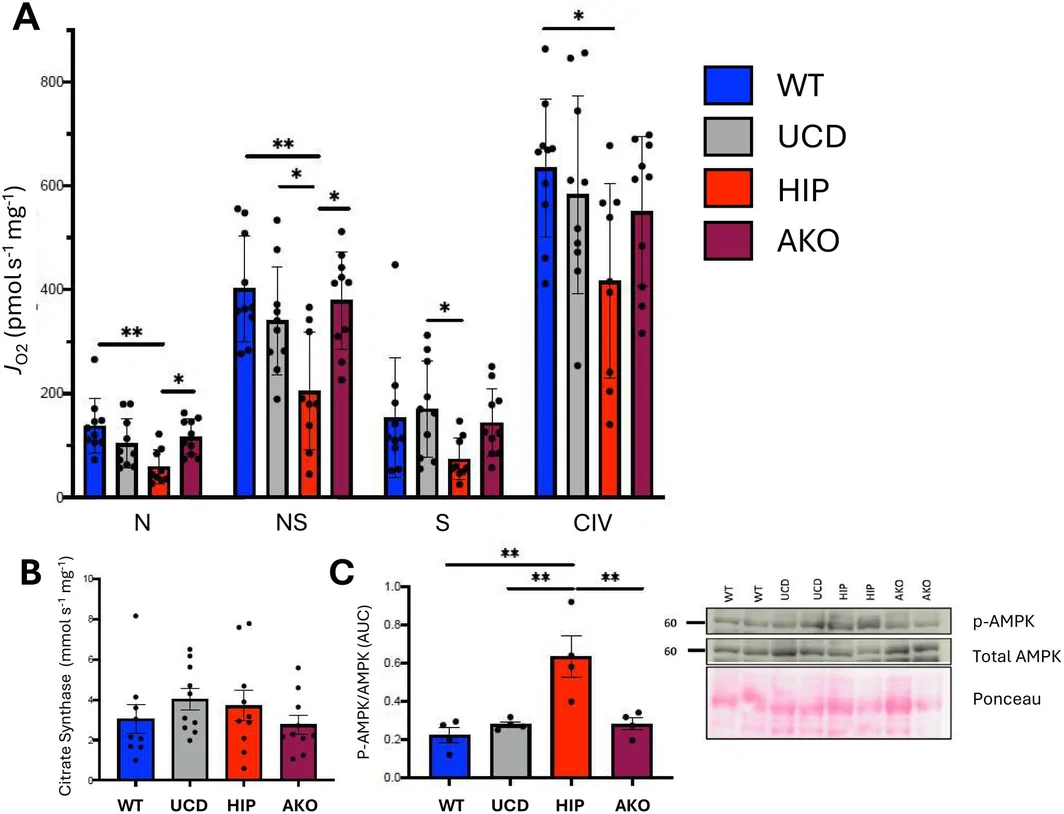
Cardiac electron transfer system (ETS) and citrate synthase activity, and AMPK activation. (A) Oxygen flux (*J*_O2_) in heart homogenates from wild‐type (WT), hyperglycemic (UCD), human amylin‐expressing (HIP) and amylin‐knockout (AKO) rats. Fluxes measured in the presence of NADH supporting complex I (N), NADH and succinate supporting complex I and II (NS), succinate with rotenone supporting complex II (S) and TMPD with ascorbate supporting complex IV (*n* = 9–10 per group). (B) Cardiac citrate synthase activities (*n* = 9–10 per group). (C) Ratio of p‐AMPK to total AMPK in cardiac homogenates (*n* = 4 per group), alongside representative blot and Ponceau stain. One‐way ANOVA with post hoc Tukey's multiple comparison test. Data represented as mean ± SEM. *p < 0.05, **p < 0.01.

In HIP rats, myocardial respiratory capacities for the N‐pathway via complex I (supported by NADH), the NS‐pathway via complexes I&II (supported by NADH and succinate) and complex IV capacity (supported by ascorbate and TMPD, and corrected for auto‐oxidation using sodium azide) were 57.3% (*p* < 0.01), 49.0% (*p* < 0.01), and 34.7% (*p* < 0.05) lower respectively, compared with WT rats (Figure 6A). Additionally, NS‐linked and S‐linked respiratory capacities were 39.7% (*p* < 0.05) and 56.5% (*p* < 0.05) lower respectively in HIP rat hearts compared with UCD rat hearts. N‐linked and NS‐linked respiratory capacities were 54.5% (*p* < 0.05) and 51.3% (*p* < 0.05) lower in HIP rat hearts compared with those of AKO rats. There were no significant differences in ETS capacity in any state between WTs, UCDs, and AKOs (Figure 6A).

These results therefore suggest that amyloid‐forming amylin, secreted by the pancreas of HIP rats, was associated with impairments in mitochondrial ETS capacities in the hearts of 14–16 month rats compared with wild‐type rats. These alterations were not seen in blood glucose‐matched UCD rats or AKO rats, so could not be explained by hyperglycemia or the presence or absence of native rodent amylin. These results were associated with increased myocardial HIF‐1α and HIF‐2α, and align with previous findings of impaired ETS capacity in fresh myocardial tissue preparations from hypoxic rats (Ashmore et al. 2014; Horscroft et al. 2015). There were, however, no differences in myocardial citrate synthase activities (Figure 6B) or levels of representative ETS subunits (Figure S2A–E) between any groups, and as such the ETS impairments in HIP rats appear not to be caused by a lower mitochondrial content or altered ETS protein expression.

To further understand these observed differences in ETS capacity, myocardial respiration rates for all rats were expressed relative to citrate synthase activity from the same hearts (Figure S3). When normalized to citrate synthase, respiratory capacities supported by the N‐pathway (*p* < 0.05) and the NS‐pathway (*p* < 0.05) were lower in HIP rats compared to WT rats (*p* < 0.05), suggesting a specific, intrinsic suppression of complex I‐supported respiration in the hearts of these rats.

To understand whether amylin aggregation, HIF‐2α stabilization and ETS impairment might be associated with alterations in cardiac energetic status in the hearts of HIP rats, the ratio of phosphorylated to total AMPK (p‐AMPK/AMPK) was measured using immunoblotting. In the hearts of HIP rats, p‐AMPK/AMPK was 185.7% greater than in WT rat hearts (*p* < 0.01), 127.2% greater than in UCD rats (*p* < 0.01), and 125.4% greater than in AKO rats (*p* < 0.01; Figure 6C). Therefore, in comparison with the hearts of age‐matched rats in other groups, the HIP rat myocardium exhibited elevated markers of energetic stress.

## 4 Discussion

### 4.1 Key Findings

In this study, we sought to investigate whether deposits of amyloid‐forming amylin in the livers and hearts of HIP rats promoted tissue hypoxia signaling during the development of T2D, and if this was associated with mitochondrial alterations in these tissues. We found greater accumulation of HIF‐1α and HIF‐2α in the livers of HIP rats compared with other groups, alongside increased expression of the HIF‐1 target gene *Vegfa*. Mitochondrial ETS capacity was also elevated in these livers, in conjunction with the formation of mitochondrial supercomplexes. Amylin aggregates formed in the hearts of HIP rats, alongside accumulation of HIF‐1α and HIF‐2α. This was associated with suppression of ETS capacity and increased p‐AMPK/AMPK, which may indicate an energetic impairment in these hearts.

### 4.2 Study Design

A major strength of this work lies in the animal models used, allowing us to isolate the effects of amyloidogenic amylin, whilst controlling for any impact of rodent amylin or other aspects of T2D pathology. The HIP rats overexpress human amylin, specifically from the pancreas, within a physiological range, that is, 3‐fold higher than rodent amylin. Whilst wild‐type rats (expressing only rodent amylin at normal levels) were included as controls, the additional inclusion of UCD rats, matched to HIP rats for glycemic status, allowed us to compare the impact of amyloidogenic amylin during T2D pathogenesis in HIP rats with a similar progression of T2D in the presence of rodent amylin alone. Finally, the inclusion of AKO rats provided an important mechanistic control by distinguishing pathological effects attributable to amyloidogenic human amylin from those resulting from altered physiological amylin signaling. Comparison with AKO rats allowed us to determine whether activation of hypoxia signaling and mitochondrial remodeling required the presence of amyloidogenic amylin rather than simply reflecting changes in endogenous amylin biology.

In this study, older animals (14–16 months) were used, as we were interested in understanding advanced stages of T2D, when pathological amylin deposition was established. For this reason, only male rats were used, since female HIP rats show some resistance to disease onset, developing T2D much later. It would, however, be of interest to understand whether tissue hypoxia is a feature of earlier stages of the pathology, and whether it might precede amylin deposition.

The use of frozen tissue is both a strength and limitation of this work. The development of techniques for the measurement of respiratory capacity in frozen tissues (Acin‐Perez et al. 2020; Knapton et al. 2026) allows for the use of archived samples from well‐characterized animals, including valuable samples collected from aged cohorts such as those studied here. Accordingly, this approach eliminates the need to use new animals, maximizing the return of data from human‐relevant models of advanced metabolic disease. Freeze‐thawing disrupts the integrity of mitochondrial membranes; however, ETS complexes and supercomplexes remain intact in freeze‐thawed preparations (Acín‐Pérez et al. 2008), and previous work demonstrated that reconstitution of the ETS was feasible with the supplementation of exogenous cytochrome *c*, with NADH included as an electron donor to complex I, in place of glutamate/malate (Acin‐Perez et al. 2020). We subsequently optimized this technique for the high‐resolution respirometry platform and measurement of ETS capacities in rat liver and heart samples (Knapton et al. 2026). Importantly, we validated our optimized method by assessing ETS capacities in homogenates of frozen liver and heart from normoxic and hypoxia‐exposed rats (10% O_2_ for 2 or 14 days). We thereby replicated hypoxia‐induced differences in respiratory capacity measured previously, using conventional, fresh tissue preparations (liver homogenates and saponin‐permeabilized cardiac fiber bundles) from the same rats (Knapton et al. 2026). This technique is therefore appropriate for the measurements reported here; however, the analysis of mitochondrial respiration in frozen samples is inherently more limited in scope than in fresh tissue samples as some aspects of mitochondrial metabolism (pyruvate/fatty acid oxidation, TCA cycle) cannot be measured. Moreover, it should also be noted that in this assay, substrate oxidation is uncoupled from ADP phosphorylation, and as such, coupling efficiency cannot be assessed.

### 4.3 Results in Context and Implications

Our work highlights a novel aspect of metabolic disease pathogenesis, whereby dysregulation of human amylin, secreted from the pancreas, promotes tissue hypoxia signaling in key metabolic organs, along with tissue‐specific mitochondrial alterations which are consistent with previous findings in the livers and hearts of rats exposed to inhalation hypoxia.

The liver displays a steep oxygen gradient with partial pressures of oxygen falling from relatively high levels in the periportal regions towards lower oxygenation in perivenous regions (Kietzmann 2019). This gradient is vulnerable to perturbation across disease states (Kietzmann 2019), and tissue hypoxia is a prominent feature of MASLD (Fuster‐Martínez et al. 2026), a condition closely associated with obesity and T2D (Younossi et al. 2025). HIF‐1α and HIF‐2α accumulate in the livers of MASLD patients (Morello et al. 2018; Yu et al. 2020), where they regulate fatty acid oxidation and de novo lipogenesis potentially contributing towards worsening steatosis, as well as other key processes of disease progression including fibrosis and inflammation (Holzner and Murray 2021). The mechanisms contributing to hepatic HIF activation in MASLD remain incompletely understood. Chronic intermittent hypoxia (CIH) resulting from obstructive sleep apnea is common in patients with obesity (Romero‐Corral et al. 2010) and is strongly associated with greater severity of liver pathology in MASLD (Aron‐Wisnewsky et al. 2012). Rodents, however, do not spontaneously develop OSA, yet perturbation of the hepatic oxygen gradient has been observed in mice fed a HFD alongside mitochondrial alterations (Mantena et al. 2009), indicating that hepatic hypoxia can arise during disease pathogenesis independently of OSA.

Our results suggest that pathological amylin deposition may be a further trigger for induction of HIF‐signaling during the development of advanced metabolic disease. Of note, whilst both HIF‐1α and HIF‐2α accumulated in the livers of HIP rats, a predominant HIF‐1α target, *Vegfa*, was upregulated, with no difference in expression of the canonical HIF‐2α target, *Arg1*. HIF‐1α is commonly suggested to support the response to relatively short‐term hypoxic stress (< 24 h), with HIF‐2α activation characterizing more sustained exposure to hypoxia (Koh and Powis 2012). As such, this finding may not appear consistent with the sustained tissue hypoxia that occurs during long‐term metabolic disease. Our finding of enhanced hepatic ETS capacity in these rats, independent of changes in mitochondrial content, alongside upregulation of *Higd1a* and supercomplex formation, does however align with our previous findings in rats exposed to relatively short‐term hypoxic stress (2 day, 10% O_2_) (O'Brien et al. 2021). This response was found to play a critical role in supporting metabolic homeostasis during this initial period of hypoxic exposure, prior to systemic acclimation, which included increased hematocrit. We propose that the finding of a similar response in older HIP rats may reflect sustained HIF signaling in this model and a failure of systemic oxygen delivery to ameliorate this. Supporting this view, HIP rats show an elevated hematocrit owing to an exaggerated erythropoiesis, which arises secondary to activation of renal hypoxia signaling (Verma et al. 2020). Amylin‐coated red blood cells in these rats are however characterized by lower deformability and lower levels of functional (i.e., non‐glycated) hemoglobin (Verma et al. 2020), indicating that this systemic mechanism to enhance oxygen carriage capacity and convective oxygen delivery may be ineffective in offsetting tissue hypoxia signaling and downstream metabolic responses. Importantly, inclusion of the AKO rats strengthens this interpretation. If activation of hypoxia signaling merely reflected altered physiological amylin signaling, one would expect similar changes in the absence of endogenous amylin. Instead, the phenotype was most pronounced in HIP rats expressing amyloidogenic human amylin, indicating that the pathological consequences arise from the gain of amyloidogenic properties rather than from loss or perturbation of normal amylin function. Together with the UCD comparison, these findings support the conclusion that amyloid‐forming human amylin represents a distinct pathogenic driver of tissue hypoxia signaling beyond the metabolic abnormalities accompanying diabetes itself.

Myocardial hypoxia can arise in acute settings, most notably myocardial infarction, whilst more sustained hypoxia and activation of HIF signaling has been proposed to play a role in the pathogenesis of heart failure (Giordano 2005). Acutely, HIF‐1 and HIF‐2 accumulation has been reported in peri‐infarct regions of rat myocardium following myocardial infarction, alongside expression of downstream targets (Willam et al. 2006), whilst HIF‐1α levels are seen in ischemic human heart biopsies during coronary bypass surgery (Lee et al. 2000). In these contexts, HIF signaling is likely to play a protective role limiting oxidative damage and loss of viable tissue, with enhanced glycolytic flux but maintained mitochondrial capacity during shorter‐term hypoxia (Horscroft et al. 2015). In contrast, sustained hypoxia results in suppressed myocardial fatty acid oxidation and mitochondrial respiratory capacity (Ashmore et al. 2014; Cole et al. 2016; Horscroft et al. 2015, 2019), including complex I‐supported respiration (Ashmore et al. 2014; Horscroft et al. 2015) which corresponds to the intrinsic mitochondrial alterations we found in HIP rats. Whilst these metabolic alterations might serve to match oxygen demand to the diminished supply, sustained hypoxic exposure is associated with myocardial energetic impairment and altered function (Holloway et al. 2011). Aligning with these findings, our results suggest that deposition of pathological amylin is associated with sustained myocardial HIF‐2 activation and suppression of ETS capacity. Our finding of increased p‐AMPK/AMPK supports a decline in energetics in the HIP rat hearts, though further measures of energetics (e.g., myocardial ATP, or PCr/ATP) would help to confirm this. AMPK activation is itself associated with enhanced glucose utilization during myocardial ischemia (Marsin et al. 2000) and cardioprotection upon reperfusion (Russell et al. 2004).

There is evidence to suggest that HIF signaling is suppressed in the hearts of patients with T2D, and this may explain the increased susceptibility of these patients to myocardial ischemia/reperfusion injury (Heather and Clarke 2011). Lower HIF‐1α levels and *VEGFA* expression were seen in myocardial biopsies of T2D patients undergoing coronary bypass surgery compared with patients without diabetes (Marfella et al. 2004). Similarly, hyperglycemic rats with streptozotocin‐induced diabetes showed a blunted HIF‐1α response to ischemia, although notably these rats showed greater *Hif1a* expression at baseline in comparison with normoglycemic rats (Marfella et al. 2002). Whilst the mechanisms leading to a blunting of HIF‐1α activation in the diabetic heart are not fully understood, enhanced fatty acid oxidation (Dodd et al. 2018) and/or PPARα activation (Zhou et al. 2012) may play a role. Pharmacological restoration of HIF signaling in the hearts of animal models of diabetes has been demonstrated to promote glucose metabolism and have cardioprotective effects (Fialho M da et al. 2021; Xue et al. 2012). Importantly, the blunting of HIF‐1α activation may be model dependent, with work in one rat model of T2D (high‐fat diet, low‐dose streptozotocin) showing myocardial HIF activation following exposure to inhalation hypoxia, alongside enhanced glycolysis and suppression of fatty acid oxidation, although these metabolic changes were less pronounced than the responses seen in control animals (Mansor et al. 2016). Of note, the role of HIF‐2α in the diabetic heart is relatively underexplored, although multiple lines of evidence point towards a protective role for HIF‐2α during myocardial ischemia, including protection of microvascular barrier function, preservation of function (Ullah et al. 2025) and limitation of myocardial damage (Koeppen et al. 2018). Accordingly, HIF‐2α and mitochondrial suppression in the HIP rat heart may play a protective role in the context of sustained hypoxic stress.

Our findings highlight contrasting tissue‐specific effects of sustained pancreatic secretion of amyloidogenic amylin on ETS capacities in HIP rats. One potential explanation for the divergent mitochondrial responses lies in the distinct adaptive demands placed on liver and heart during sustained hypoxic stress. In the liver, HIF activation may promote adaptive mitochondrial remodeling that preserves oxidative metabolism despite reduced oxygen availability. Our finding of increased expression of the HIF target gene *Higd1a*, together with enhanced respiratory supercomplex formation, supports this interpretation. HIGD1A has been shown to stabilize respiratory chain complexes and promote supercomplex assembly under hypoxic conditions, thereby improving electron transfer efficiency and limiting oxidative stress (Hayashi et al. 2015; Timón‐Gómez et al. 2020). Although we did not examine the structural mediators of this response, it is plausible that HIF‐dependent induction of HIGD1A acts in concert with mitochondrial cristae remodeling, potentially involving OPA1‐dependent stabilization of supercomplexes, as described in other models of metabolic stress (Cogliati et al. 2013; Del Dotto et al. 2017).

In contrast, this adaptive response may be prevented in heart as a result of direct cardiomyocyte toxicity arising from amyloidogenic amylin, impairing substrate oxidation and mitochondrial function. Human amylin aggregates have been shown to accumulate within cardiomyocytes, disrupt mitochondrial membrane integrity, impair oxidative phosphorylation, and promote energetic stress (Despa et al. 2012; Liu et al. 2016). These mechanisms are consistent with the reduced ETS capacity and increased AMPK activation observed in HIP rat hearts. Rather than representing contradictory consequences of HIF activation, the divergent mitochondrial phenotypes may therefore reflect tissue‐specific remodeling, whereby the liver adapts to sustained hypoxic stress by reorganizing the respiratory chain, whereas the heart develops a maladaptive energetic phenotype driven by the combined effects of hypoxia and amylin proteotoxicity.

The differing mitochondrial responses in heart and liver might in turn have contrasting implications for metabolic homeostasis and function, though they may instead represent appropriate context‐specific responses to hypoxia across these two tissues. The healthy heart is highly dependent on oxidative phosphorylation to meet the high ATP demands associated with contractile function. Accordingly, in the chronically‐hypoxic human heart, myocardial energetic reserve falls (measured as a lower ratio of phosphocreatine‐to‐ATP (PCr/ATP) using ^31^P‐NMR spectroscopy) and this coincides with impaired myocardial filling during diastole (Holloway et al. 2011). Similarly, in hypoxic mouse hearts, suppression of oxidative metabolism including fatty acid oxidation was associated with a diminished PCr/ATP, alongside greater end diastolic volumes and diminished stroke volumes in these mice, but preserved ejection fraction and cardiac output (Cole et al. 2016). Of note, in hypoxic mice fed a high fat diet, which resulted in restoration of oxidative metabolism, PCr/ATP remained low and functional parameters were worsened in comparison with hypoxia alone, including a lower cardiac output (Cole et al. 2016). This suggests that in the context of severe and sustained hypoxia, where a fall in energetic reserve is unavoidable, suppression of myocardial mitochondrial respiration is protective of contractile function (Cole et al. 2016). In contrast, the liver exhibits steep hypoxic gradients in the healthy state and has a lower reliance on oxidative metabolism than heart. Here, enhanced ETS capacity above a relatively low baseline may serve to maintain metabolic function. Indeed, during short‐term exposure to environmental hypoxia, an increased respiratory capacity associated with supercomplex formation occurred in rat liver, alongside maintained energetic homeostasis (O'Brien et al. 2021). Strikingly, treatment of these rats with a complex III inhibitor that repressed the enhanced ETS capacity resulted in hepatic metabolic dysregulation and loss of energetic homeostasis (O'Brien et al. 2021). Collectively, these findings therefore suggest that the differing impacts of amyloidogenic amylin on ETS capacities in the heart and liver of HIP rats might represent appropriate tissue‐specific mitochondrial responses occurring downstream of HIF activation. Accordingly, this would suggest that prevention of amylin aggregation itself and the tissue hypoxia that may ensue might represent a more viable treatment strategy than directly targeting the downstream mitochondrial response.

Emerging evidence (Davargaon et al. 2026; Ilaiwy et al. 2016; Verma et al. 2023) identifies (pre)diabetic amylin hypersecretion as a systemic stressor that converges on shared signaling and metabolic pathways across peripheral organs and the central nervous system. In both human Alzheimer's disease brains and HIP rats, aggregated amylin deposits in cerebral microvessels (Ly et al. 2021; Verma et al. 2023) compromise perfusion and oxygen delivery and alter hypoxia‐ and metabolism‐related gene expression (Verma et al. 2023). In parallel, studies in amylin‐humanized mice exposed to high‐fat diet‐induced metabolic stress show amylin‐driven cAMP‐PKA overactivation that further exacerbates mitochondrial dysfunction (Davargaon et al. 2026). Elevated PKA activity increases neuronal energy demand via enhanced ion channel activity and neurotransmitter cycling (Dridi et al. 2023), whilst impairing mitochondrial performance through aberrant phosphorylation of glycolytic enzymes (Davargaon et al. 2026). This dual effect creates a mismatch between energy demand and supply, analogous to the energetic impairment observed here in the heart. Our working hypothesis is that amylin‐induced microvascular dysfunction initiates tissue hypoxia and mitochondrial stress, activating compensatory yet maladaptive pathways (including HIF and AMPK), sustaining cAMP‐PKA overactivation and disrupting insulin signaling and glucose utilization. This interpretation is supported by findings that amylin oligomers circulate through the blood and accumulate in the heart (Despa et al. 2012; Jackson et al. 2012), kidney (Verma et al. 2020), and brain (Ly et al. 2021; Verma et al. 2023). Amylin accumulation in tissue is associated with endothelial dysfunction (El Assar et al. 2015; Novials et al. 2007), impaired microvascular integrity (Verma et al. 2020) and inflammatory responses (Liu et al. 2016; Verma et al. 2020). These interconnected pathological processes across liver, heart and brain suggest that systemic amylin dyshomeostasis drives multi‐organ metabolic failure through mechanisms involving hypoxia signaling, mitochondrial remodeling and dysregulated cAMP‐PKA activity. The four experimental groups facilitate separation of the effects of diabetes, physiological amylin signaling, complete amylin deficiency, and amyloidogenic human amylin, thereby providing stronger evidence that the observed hypoxia signaling and mitochondrial remodeling are specifically associated with the amyloidogenic properties of human amylin rather than diabetes per se. Our current data, however, do not allow us to distinguish whether the observed effects are driven primarily by: (i) local tissue deposition of aggregated amylin; (ii) circulating soluble or aggregated amylin acting systemically; or (iii) activation of amylin receptors on resident cells. Further, whilst HIF activation may be indicative of amylin‐mediated tissue hypoxia, it is important to note that HIF prolyl hydroxylation (signaling HIF degradation) can be inhibited by reactive oxygen species and TCA cycle intermediates (succinate, fumarate), resulting in HIF stabilization in the absence of a fall in tissue P_O2_. As such, the observed HIF activation is consistent with, but does not establish, a mechanism involving microvascular dysfunction and tissue hypoxia.

### 4.4 Further Work

Future studies combining tissue amylin localization, quantitative assessment of organ microvascular architecture and perfusion, endothelial injury markers, fibrosis, and pharmacological or genetic inhibition of amylin receptor signaling will be required to determine the relative contributions of tissue deposition, circulating amylin, and receptor‐mediated signaling to organ dysfunction. One key unanswered question arising from this work concerns the mechanism(s) leading to HIF activation in the HIP rat liver and heart, and whether this is driven by altered oxygen delivery owing to RBC and/or microvascular dysfunction, amylin deposition in or around hepatocytes/cardiomyocytes or a signaling effect of human amylin resulting in HIF stabilization. Although not statistically significant, we observed a trend for higher HIF‐2α in the livers of UCD rats (which hypersecrete rodent amylin) in comparison with AKO rats (Figure 1B), possibly hinting at more direct interactions between amylin signaling and the HIF pathway. A potential signaling role for amylin could be explored further in cultured cell preparations, which could include the use of amylin receptor (AMYR) antagonists. Meanwhile the sequence of events relating to amylin deposition, HIF activation and mitochondrial alterations could be better elucidated by studying rats at an earlier stage of disease progression, and such work could include more direct measures of tissue hypoxia beyond HIF levels and expression of target genes, for example, pimonidazole hydrochloride staining. Mechanisms connecting amyloidogenic amylin hypersecretion to end‐organ metabolic alterations could be further explored via use of the Conditional Amylin Knock‐In (CAKI) mouse, which has insertion of a LoxP human amylin gene (HuAmy) expressed specifically in the pancreas, alongside pancreatic specific Ins‐1 CreER insertion (Davargaon et al. 2026). Work with this model could help to elucidate causal links between amylin aggregation, tissue hypoxia, HIF‐activation and mitochondrial alterations, and would enable mechanistic evaluation of whether the mitochondrial responses are protective or detrimental in this context. Finally, treatment strategies known to limit/prevent amylin aggregation, for example, epoxyeicosatrienoic acids (EETs) (Jiang et al. 2010; Verma et al. 2020) or anti‐amylin immunotherapy (Kotiya and Despa 2024; Vogt et al. 2021) could be explored to understand whether such strategies prevent end‐organ HIF activation and metabolic alterations.

## 5 Conclusions

Pancreatic secretion of amyloidogenic amylin is associated with activation of hypoxia signaling in key metabolic organs beyond the renal vasculature, alongside mitochondrial alterations in liver and heart, which are themselves consistent with findings in models of hypoxic stress. Our results therefore support previous work suggesting that amylin dysregulation is an overlooked, human‐relevant aspect of diabetes pathogenesis and is, as such, a potential therapeutic target.

## Author Contributions

**Alice E. Knapton:** data curation, formal analysis, investigation, methodology, writing – original draft; **Nirmal Verma:** formal analysis, investigation, writing – review and editing; **Deepak Kotiya:** formal analysis, writing – review and editing; **Alice P. Sowton:** investigation, writing – review and editing; **Benjamin D. Thackray:** investigation, writing – review and editing; **Lorenz M. W. Holzner:** investigation, writing – review and editing; **Paula M. Darwin:** investigation, writing – review and editing; **Sanda Despa:** conceptualization, formal analysis, funding acquisition, methodology, resources, supervision, writing – review and editing; **Florin Despa:** conceptualization, data curation, formal analysis, funding acquisition, methodology, resources, supervision, writing – original draft; **Andrew J. Murray:** conceptualization, data curation, formal analysis, funding acquisition, methodology, supervision, writing – original draft.

## Funding

This work was supported by National Institutes of Health: awards R01HL135000 and R01HL148443 to S.D. and awards R01HL118474, R01AG057290, R01AG053999 and R01NS116058 to F.D. This work was also supported by PhD Studentships from the British Heart Foundation (FS/19/54/34889C to A.E.K.), (FS/17/61/33473 to A.P.S.), (FS/4yPhD/F/20/34124 to B.D.T.), a PhD studentship from the Wellcome Trust (220033/Z/19/Z to L.M.W.H.), a Biotechnology and Biological Sciences Research Council Doctoral Training Program award (BB/M011194/1 to P.M.D.), a Research Councils UK award (EP/E500552/1 to A.J.M.), and Evelyn Trust (Grant: 16/33 to A.J.M.).

## Ethics Statement

All work was performed in accordance with the NIH Guide for the Care and Use of Laboratory Animals, with prior approval by the Institutional Animal Care and Use Committee, University of Kentucky.

## Conflicts of Interest

The authors declare no conflicts of interest.

## Supporting information


**Figure S1:** Liver respiratory capacities expressed relative to citrate synthase activity.
**Figure S2:** Cardiac electron transfer system (ETS) protein levels.
**Figure S3:** Heart respiratory capacities expressed relative to citrate synthase activity.
**Table S1:** Qiagen primers used.

## Data Availability

Data is available via the University of Cambridge repository: https://doi.org/10.17863/CAM.130215. (NB: placeholder doi. Link will go live following acceptance).
