# Supplementary material for: Amyloid‐Forming Amylin Secreted by the Pancreas Promotes Hypoxia‐Inducible Factor Activation and Mitochondrial Alterations in Liver and Heart During Type 2 Diabetes Pathogenesis
Source: Compr Physiol. 2026 Sep 4;16(5):e70252. doi: 10.1002/cph4.70252 (PMC13545475; doi:10.1002/cph4.70252)
Supplement: Supplementary file 1 [file CPH4-16-e70252-s001.docx]

**Supplementary Material for**

**Amyloid-forming amylin secreted by the pancreas promotes hypoxia-inducible factor activation and mitochondrial alterations in liver and heart during type 2 diabetes pathogenesis**

Alice E Knapton, Nirmal Verma, Deepak Kotiya, Alice P Sowton, Benjamin D Thackray, Lorenz MW Holzner, Paula M Darwin, Sanda Despa, Florin Despa, Andrew J Murray

**Full Methods**

*Animals*

Animal work was performed in accordance with the NIH Guide for the Care and Use of Laboratory Animals, with prior approval by the Institutional Animal Care and Use Committee, University of Kentucky.

We compared four groups: HIP rats (n = 21), which develop T2D due to expression of amyloid-forming human amylin; UCD rats (n = 19), which develop obesity, insulin resistance, and diabetes without amyloid (expressing non-amyloidogenic rat amylin); non-diabetic wild-type controls (WT; n = 19); and amylin knockout rats (AKO; n = 12). Rats were studied at 14-16 months of age, and only males were used because HIP females become diabetic at a more advanced age (1, 7).

HIP rats (SD-Tg(Ins2-IAPP)Soel) are Sprague-Dawley (SD) rats that overexpress (3-fold) human amylin specifically in pancreatic β-cells on the insulin II promoter (1). HIP rats develop amylin dyshomeostasis and amylin amyloid deposition in pancreatic islets leading to β-cell apoptosis and hyperglycemia with regular diet. Breeding pairs were obtained from Charles River Laboratories. Presence of the human amylin gene was confirmed by genotyping using the following primers: Rip1: 5’- GTC ATG TGC ACC TAA AGG GGC AAG TAA TTC A-3’; Rip2: 5’- CGA GTG GGC TAT GGG TTT GT -3’; Mus-a-actinF1: 5’-CAC CAC ACC TTC TAC AAT GAG CTG-3’; Mus-a-actinR1: 5’-TCA TCA GGT AGT CAG TGA GGT CGC -3’. Actin was used as the internal control. WT littermates were used as non-diabetic controls.

UCD rats were generated by crossing obese SD rats with Zucker diabetic fatty (ZDF) lean rats (2). ZDF-lean founder rats were purchased from Charles River Laboratories. These rats have functional leptin receptors and the autosomal recessive β-cell defect. The breeding strategy was to maintain the obese phenotype from obese SD rats whilst selecting individuals homozygous for the β-cell defect. In the seventh generation (F7) all animals were homozygous for the β-cell defect and increased genetic propensity to develop diabetes.

AKO rats are SD rats in which rodent amylin expression has been knocked out. The rat amylin gene was targeted at two genomic sites by producing double strand breaks using CRISPR-Cas9. CRISPR gRNAs and Cas9 mRNA were injected into the pronucleus of fertilised SD rat embryos (7). Amylin KO was confirmed by genotyping using the following primers: Forward: 3’ – AGAGCTAAGCAAGTTGAGGGAT – 5; Reverse: ACCACTAGTTCACATTCACAGAGG.

Rats were single-housed in individually ventilated cages (ACE, Allentown, NJ, USA) and maintained on a standard chow diet (Teklad Global 18% Protein Rodent Diet #2018, VA, USA) and reverse osmosis drinking water for the duration of the study. All animals were maintained on a 12:12 hour light:dark cycle at temperatures between 21-24 ^º^C.

Rats were culled by a single terminal surgical excision of the heart whilst under deep isoflurane anaesthesia (3-5% isoflurane in 100% oxygen). Adequate depth of anaesthesia was verified by a lack of reflex upon toe pinch. Organs were collected using sterile surgical instruments and embedded in paraffin or snap-frozen in liquid nitrogen before being stored at -80°C.

Rats in the four groups were matched by age (WT – 16.4 ± 0.3 months, HIP – 15.1 ± 0.4 months, UCD – 14.5 ± 0.3 months, and AKO – 15.1 ± 1.7 months). The diabetic HIP and UCD rats were also matched by blood glucose levels at the time of euthanasia (505 ± 23 mg/dL and 415 ± 42 mg/dL, respectively).

*Respirometry*

Respirometry was performed on homogenates prepared from previously-frozen liver and heart to assess electron transfer system (ETS) capacity, using a published protocol (6).

Snap-frozen liver (~15 mg) was defrosted in ice-cold BIOPS (2.77 mM CaK_2_EGTA, 7.23 mM K_2_EGTA, 6.56 mM MgCl_2_·6H_2_O, 20 mM taurine, 15 mM sodium phosphocreatine, 20 mM imidazole, 0.5 mM dithiothreitol (DTT), 50 mM K-2-(N-morpholino)ethanesulfonic acid (MES), 5.77 mM Na_2_ATP, pH 7.1). Tissue was weighed, blotted, then transferred to ice-cold MiR06 (0.5 mM EGTA, 3 mM MgCl_2_·6H_2_O, 60 mM K-lactobionate, 20 mM taurine, 10 mM KH_2_PO_4_, 20 mM HEPES, 110 mM sucrose, 1 g·L^−1^ defatted bovine serum albumin (BSA), pH 7.1, with 280 U.mL^-1^ catalase) at a volume yielding a 10% (w/v) homogenate. Homogenisation was carried out using an Eppendorf pestle and DLH overhead stirrer (Velp, Scientifica) with short pulses (~5 s) at 600 rpm. Homogenate was added to pre-warmed MiR06 in Oxygraph chambers (37°C) at a concentration of 1 mg.mL^-1^. *J*_O2_ was initially measured following addition of cytochrome *c* (10 μM) to reconstitute the ETS, and superoxide dismutase (SOD; 5 U.mL^-1^). Malate (1 mM) and pyruvate (25 mM) were added to confirm limited TCA cycle operation owing to loss of mitochondrial membrane integrity post-freeze thawing. Next the N-pathway via complex I was stimulated with NADH (1.25 mM; N-linked), followed by succinate to additionally stimulate the S-pathway via complex II (10 mM; NS-linked). Complex I was inhibited by rotenone (2.5 µM), allowing assessment of S-pathway dependent ETS capacity (complex II only; S-linked). Antimycin A (5 µM) was added to inhibit complex III, isolating electron flux through complex IV which was assessed using ascorbate (2 mM) and TMPD (0.5 mM). These substrates undergo autoxidation, and so *J*_O2_ was measured (at an O_2_ concentration of 200 µM), before chambers were reoxygenated and sodium azide (100 mM) added to inhibit complex IV. *J*_O2_ was again recorded at an O_2_ concentration of 200 µM and deducted from the previous rate to determine complex IV-dependent O_2_ consumption.

Snap-frozen cardiac samples (~5 mg) were defrosted in ice-cold BIOPS, transferred to 500 µL ice-cold MiR06 (as above, but with 400 U.mL^-1^ catalase) and minced with fine scissors. Tissue was homogenised using a pre-chilled PBI-Shredder SG3 (Oroboros Instruments), at Level 1 for 10 s and Level 2 for 5 s. Homogenate was removed and the Shredder-tube flushed with 4.5 mL MiR06. Volume was adjusted to give a final concentration of 1 mg.mL^-1^, and the homogenate transferred to Oxygraph chambers (37°C). Cytochrome *c* (10 µM) was added to Oxygraph chambers to reconstitute the ETS, and SOD (25 U.mL^-1^) and S1QEL1.1 (0.4 µM) added to suppress superoxide production at site I_Q_ at complex I, without inhibiting forward ETS flux. Malate (1 mM) and pyruvate (25 mM) were added to confirm TCA cycle loss. The N-pathway via complex I (N-linked) was stimulated with NADH (4.4 mM), followed by succinate (12.5 mM) to stimulate the S-pathway via complex II, and reveal maximal ETS capacity (NS-linked). Complex I was inhibited with rotenone (0.5 µM), revealing S-pathway capacity via complex II (S-linked). Antimycin A (5 µM) was added to inhibit electron flux via complex III, and complex IV capacity assessed using ascorbate (2 mM) and TMPD (0.5 mM) with auto-oxidation corrected following addition of sodium azide (100 mM, as described for liver).

*Immunohistochemistry*

Paraffin-embedded heart blocks (WT, UCD and HIP rats) were cut into 5 µm thick sections. Tissue sections were then deparaffinized in xylene, and rehydrated two times in serial dilutions (100%, 95%, and 70%) of alcohol to 1x PBS. Sections were incubated in 1x retrieval buffer (S1699; Dako) for 30 min at 95°C in a steamer for antigen retrieval. Sections were blocked in 15% horse serum and then incubated overnight with an anti-amylin primary antibody (SC-377530, Santa Cruz; 1:200 dilution). After washing, sections were incubated with biotinylated goat anti-mouse IgG secondary antibody (BA2000, Vector lab; 1:400 dilution), followed by incubation with HRP conjugated avidin-biotin complex (ABC kit; PK-6100; Vectastain laboratories, CA), and then developed in 3-amino-9-ethylcarbazole (AEC; K3461; Dako; CA) chromogen according to the manufacturer’s protocol. Sections were again blocked in 10% normal goat serum and incubated overnight with an anti-HIF-2α antibody (ab109616, abcam; 1:100 dilution) followed by an AP conjugated anti-rabbit IgG secondary antibody (A3687, Sigma; 1:50 dilution). Then, sections were developed in StayGreen/AP Plus (ab156428; abcam) chromogen according to manufacturer’s protocol. Finally, sections were mounted in aqueous mounting media and imaged with a Nikon Eclipse 55i upright microscope.

*Immunofluorescence*

Heart sections were prepared as described in the Immunohistochemistry section (up to the antigen retrieval step). Sections were then blocked for 1 h in a solution of 10% goat serum, 5% BSA, and 0.5% Triton X-100 and washed once in PBS. Sections were incubated with an anti-human amylin antibody (T-4157, Bachem-Peninsula Laboratories; 1:200 dilution) overnight at 4°C, then washed three times for 5 min with 0.1% Triton in Tris-NaCl. Sections were incubated with Alexa Fluor 488 conjugated anti-rabbit IgG secondary antibody (A-11008; Invitrogen; 1:500 dilution) for 1 h. The sections were again washed three times for 5 min with 0.1% Triton in Tris-NaCl and autofluorescence was blocked in 0.1% Sudan black (BP109-10, Fisher) for 10 min and washed in 1x PBS thrice. They were cover-slipped in mounting media with DAPI (ab 104139, Abcam; MA) and imaged with a confocal microscope.

*HIF-1α and HIF-2α ELISAs*

For the detection of HIF-1α and HIF-2α in tissue homogenates, Rat Hypoxia Inducible Factor 1 Alpha (HIF-1α) or Rat Hypoxia Inducible Factor 2 Alpha (HIF-2α) ELISA kits (MyBioSource) were used according to manufacturer’s instructions.

Tissue samples were homogenised in HIF lysis buffer using steel balls and a tissue lyser (Qiagen) for 2 min at 20 Hz. Homogenate was centrifuged using a PrismR centrifuge (Labnet International) at 10,000 rpm for 10 min, 4°C. Protein concentration was then determined using the Pierce BCA Protein Assay kit (Thermo Scientific). Following trial runs, samples were diluted in HIF lysis buffer to obtain 50 μg of protein per sample. For each sample, 50 μL was loaded in duplicate into plate wells (from kit). HIF standards were made up using 1:2 serial dilution steps from the kit standard (10 ng.μL^-1^) diluted in standard diluent. Blank samples contained only standard diluent. 100 μL of each standard was plated in duplicate before the plate was sealed and incubated at 37°C for 90 min. The ELISA plate was washed twice. 350 μL of wash buffer was added per well and left to sit for 30 sec before thoroughly removing each time. The biotinylated antibody (in kit) was prepared in antibody diluent (1:100) before 100 μL was added to every well. The plate was sealed and incubated at 37°C for 60 min. The ELISA plate was washed three times as above. The enzyme conjugate (in kit) was prepared in enzyme diluent (1:100) before 100 μL was added to every well, except the blanks. The plate was sealed and incubated at 37°C for 30 min. The ELISA plate was washed five times as above. Colour Reagent (in kit) was prepared using Reagent A and B (9:1) before 100 μL was added to every well. The ELISA plate was incubated at 37°C under foil for 10 min. 100 μL Colour Reagent C (in kit) was added to each well. The plate was read at 450 and 630 nm within 10 min using a FLUOstar Omega plate reader (BMG Labtech).

*Amylin ELISA*

A competitive ELISA assay kit (EIA-AMY-1, Ray-biotech) was used to measure the amylin content in whole heart homogenates from age matched male WT, HIP and UCD rats. The protocol followed manufacturer’s instructions.

*Enzyme activity analysis*

Tissue homogenates were generated by adding a small section of snap-frozen tissue to 300 μL homogenisation buffer (20 mM HEPES, 1 mM EDTA, 0.1% Triton X-100, pH 7.2) and homogenised using a liquid nitrogen-cooled Eppendorf pestle inserted into an overhead stirrer at <600 rpm, with 3 rapid strokes (Velp Scientifica Overhead Stirrer). The homogenate was centrifuged at 380 x *g* for 30 sec, 4°C. Supernatant was extracted and re-spun before the final supernatant was taken. Protein concentration was quantified using the Pierce BCA Protein Assay kit (Thermo Scientific) and a microplate reader (Elx800, BioTek).

Citrate synthase activity was assayed as previously described (4). A volume of homogenate containing 10 μg protein was made up to 925 μL with citrate synthase assay buffer (20 mM Tris-Base, 0.1 mM 5,5-dithiobis-2-nitrobenzoic acid (DTNB), pH 8.0). Following this, 0.6 mM of acetyl CoA was added, and the mixture was warmed at 37°C for 3 min, before baseline absorbance was read on a spectrophotometer at 412 nm for 3 min. Oxaloacetate (0.5 mM) was then added to result in the following reaction, catalysed by citrate synthase:

𝐴𝑐𝑒𝑡𝑦𝑙 𝐶𝑜𝐴𝑆𝐻 + 𝑂𝑥𝑎𝑙𝑜𝑎𝑐𝑒𝑡𝑎𝑡𝑒 + 𝐻_2_𝑂 → 𝐶𝑜𝐴𝑆𝐻 + 𝐶𝑖𝑡𝑟𝑎𝑡𝑒

This is coupled to the following reaction:

𝐶𝑜𝐴𝑆𝐻 + 𝐷𝑇𝑁𝐵 → 𝑇𝑁𝐵 + 𝐶𝑜𝑆𝑆𝑇𝑁𝐵

The production of thionitrobenzoic acid (TNB) is therefore dependent on the activity of citrate synthase in the sample. TNB absorbs at 412 nm, allowing for the rate of change of absorbance to be recorded over the course of the reaction. Baseline rate was subtracted from the reaction rate to calculate the enzyme-dependent rate of absorbance change. Enzyme activity was calculated as described previously (3).

*Immunoblotting*

Frozen tissue was disrupted and homogenised in lysis buffer (20 mM Tris-HCl pH 7.5, 1% Triton X-100, 1 mM EGTA, 150 mM NaCl, 2.5 mM Na_4_O_7_P_2_.10H_2_O, 1 mM β-glycerophosphate, 10 mM Na_3_VO_4_, protease inhibitor tablets), at 1 mL per 100 mg of tissue, using chilled steel beads and a Tissue Lyser (Qiagen) for 2 min at 20 Hz. Homogenate was centrifuged using the PrismR centrifuge (Labnet International) at 10,000 rpm for 10 min, 4°C. Protein concentration was then determined using the Pierce BCA Protein Assay kit (Thermo Scientific).

Samples were then diluted in Lysis Buffer to give 15 μL aliquots containing 25 μg protein. LDS Sample Buffer (4X, pre-diluted 1:2 in dH_2_O) and β-mercaptoethanol, at 2.5% of total volume, were added before 15 μL was loaded into 4-12% Bis Tris gels in the XCell SureLock Mini-Cell Electrophoresis System. MOPS running buffer was added, and proteins separated by running for 2.5 h at 100 V. Proteins were transferred onto PVDF membranes using a TE77 PWR Semi-Dry Transfer Unit. The gel was equilibrated, and filter papers soaked in transfer buffer. Ponceau staining was performed to enable total protein quantification in each sample lane.

*OXPHOS immunoblotting*

The membrane was blocked for 1 h at room temperature in 5% BSA, TBS-Tween (TBS-T), before incubation with the Total OXPHOS primary antibody for 1 h at room temperature (MitoProfile Total OXPHOS Blue Native Antibody Cocktail, Abcam, Cambridge, UK; 1:1000 in 5% BSA in TBS-T). After washing with 3 rounds of TBS-T for 5 min each, the membrane was incubated with an appropriate secondary antibody for 30 min at room temperature (Rb anti-ms IgG Secondary Antibody HRP conjugate, Invitrogen; 1:20,000 in TBS-T). A further three rounds of washing with TBS-T, each for 5 min, took place. Detection involved incubation with chemiluminescent horseradish peroxidase substrate (Pierce ECL Western Blotting Substrate, Life Technologies, Paisley, UK) for 5 min before imaging using Amersham Hyperfilm ECL film (Cytiva) and X-ray developer Fujifilm FPN-100.

*AMPK/p-AMPK western blotting*

The membrane was blocked for 1 h at room temperature in 5% BSA, TBS-T, before incubation with p-AMPK primary antibody overnight at 4°C (p-AMPKalpha, Cell Signalling, Technology, 1:1000 in 5% BSA in TBS-T + 0.02% Azide + 50mM NaF). After washing with 3 rounds of TBS-T for 5 min each, the membrane was incubated with a secondary antibody for 30 min (Rb anti-ms IgG Secondary Antibody HRP conjugate, Invitrogen; 1:20,000 in TBS-T). A further three rounds of washing with TBS-T, each for 5 min, took place. Detection involved incubation with chemiluminescent horseradish peroxidase substrate (Pierce ECL Western Blotting Substrate, Life Technologies, Paisley, UK) for 5 min before imaging using Amersham Hyperfilm ECL film (Cytiva) and X-ray developer Fujifilm FPN-100. Following this the membrane was placed in stripping buffer (15 g Glycine, 1 g SDS, 1% Tween-20, pH 2.2, into 1L dH_2_O) for 2 x 10 min. The membrane was re-probed for total AMPK primary antibody overnight at 4°C (AMPKalpha, Cell Signalling Technology, 1:1000 in 5% BSA in TBS-T + 0.02% Azide). The following steps were as detailed above.

*BN-PAGE*

This protocol for isolating and staining for mitochondrial supercomplexes was based on a previous protocol using small gels (5).

Snap-frozen liver (18-25 mg) was defrosted in 600 μL isolation buffer (10.269 g sucrose, 0.1815 g Tris, 1.5 mL EGTA-Tris (3.8 g EGTA in 100 mL dH_2_O, pH 7.4 using Tris), 1 protease inhibitor tablet per 50 mL). Tissue was homogenised using a liquid nitrogen-cooled Eppendorf pestle inserted into an overhead stirrer at <600 rpm, with 3 quick strokes (Velp Scientifica Overhead Stirrer). Further isolation buffer was added to the homogenate at a final adjusted volume of 0.067 mL.mg-1 tissue. Homogenate was centrifuged at 600 x *g* for 10 min, 4°C. Supernatant was re-centrifuged at 7000 x *g* for 10 min, 4°C. The pellet was re-suspended in 1.5 mL of isolation buffer and gently reconstituted, before being centrifuged again at 7000 x *g* for 10 min, 4°C. The pellet was resuspended in 250 μL of isolation buffer to give the isolated mitochondrial fraction. Protein concentration was then determined using the Pierce BCA Protein Assay kit (Thermo Scientific). Samples were diluted in isolation buffer to give aliquots containing 50 μg protein.

Aliquots were centrifuged at 7000 x *g* for 10 min, 4°C. The pellet was then re-suspended in 20 μL of Sample Cocktail Buffer (5 μL NativePAGE 4 X Sample Buffer, Invitrogen, 8 μL 5% digitonin, Invitrogen, 7 μL dH_2_O) and incubated rocking on ice for 20 min. Samples were centrifuged again at 20,000 x *g* for 10 min, 4°C , and 15 μL of supernatant was collected and reconstituted with 2 μL of NativePAGE 5% G-250 Sample Additive (Invitrogen). 15 μL of sample was loaded into 3-12% NativePAGE Bis-Tris, Mini Protein gels (NuPAGE, Novex, Invitrogen) set up within XCell SureLock Mini-Cell Electrophoresis System (Invitrogen). 15 μL of NativeMark Unstained Protein Standard (Invitrogen) was also loaded. The inner chamber of the electrophoresis tank system was filled with dark blue cathode buffer (0.044 g Coomassie Brilliant Blue G-250 in 220 mL NativePage Running Buffer), and the outer chamber with 1X NuPage running buffer (50 mL 20X NativePage running buffer in 950 mL dH_2_O), before being placed in ice, on a stirrer plate. Proteins were separated through running at 150 V for 30 min. The dark blue cathode buffer in the inner chamber was removed and replaced with light blue cathode buffer (20 mL dark blue cathode buffer into 180 mL NativePage running buffer) before running was continued at 250 V for 60 min. Band identification was achieved through immunoblotting. Analysis was performed using gels stained with Colloidal Blue.

*Colloidal blue staining*

Gels were removed from the apparatus and immersed in dH_2_O for 5 min before being incubated in 100 mL fixing solution (40 mL dH_2_O, 50 mL methanol, 20 mL acetic acid) for 10 min at room temperature. This was replaced with staining solution (55 mL dH_2_O, 20 mL methanol, 20 mL Stainer A) and incubated for 10 min at room temperature before 5 mL Stainer B was added. Gels were rocked at room temperature for 3 hours before being placed in dH_2_O overnight.

*Antibody staining*

Proteins were transferred onto polyvinylidene difluoride (PVDF) membranes (Millipore, Watford, UK) that had been activated in methanol using TE 77 PWR Semi-Dry Transfer Unit (Cytiva). Prior to transfer, the gel was equilibrated in transfer buffer (NuPAGE Transfer Buffer, Novex, Life Technologies) for 15 min. 3 filter papers, also soaked in transfer buffer (50 mL 20X NuPage running buffer (Life Technologies) to 950 mL dH_2_O), were placed into the transfer system, followed by the PVDF membrane. The gel was then carefully placed on top before 3 more soaked filter papers were stacked on top. Transfer took place over 2 h at 400 mA. Following transfer, the PVDF membrane was washed in 8% acetic acid for 5 min, followed by 2 sets of 5 min washes in 100% methanol, and 3 sets of 5 min washes in dH_2_O. The membrane was blocked for 1 h at room temperature in 5% BSA, TBS-T, before incubation with one of the following primary antibodies overnight at 4°C (NDUFA9 (complex I), SDHA (complex II), UQRC2 (complex III), MTCO1 (complex IV) (Abcam, Cambridge, UK), all 1:1000 in 5% BSA in TBS-t). After washing with 3 rounds of TBS-T for 5 min each, the membrane was incubated with an appropriate secondary antibody for 30 min at room temperature (Hu anti-ms IgG Secondary Antibody HRP conjugate, Invitrogen; 1:20,000 in TBS-T. A further three rounds of washing with TBS-T, each for 5 min, took place. Detection involved incubation with chemiluminescent horseradish peroxidase substrate (Pierce ECL Western Blotting Substrate, Life Technologies, Paisley, UK) for 5 min before imaging using Amersham Hyperfilm ECL film (Cytiva) and X-ray developer Fujifilm FPN-100.

*Protein carbonylation*

Carbonyl content was determined using a Protein Carbonyl Content Assay Kit (Abcam) according to the manufacturer’s instructions. Cardiac samples were homogenised in homogenisation buffer using steel balls and a tissue lyzer. Total protein was quantified using BCA assay kit. Homogenates were diluted with dH_2_O to give 0.5 mg of protein per sample in 150 µL. Nucleic acids needed to be removed as they interfere with the assay. To do this, samples were incubated with 15 µL of streptozotocin for 15 min at room temperature. Samples were centrifuged at 17,200 x *g* for 5 min and the supernatant was transferred to a new Eppendorf. 150 µL of 2,4-Dinitrophenylhydrazine (DNPH) was added to each sample before vortexing and incubating for 10 min at room temperature. 45 µL of Trichloroacetic acid (TCA) was then added to each sample before vortexing and incubating on ice for 5 min. Samples were centrifuged at 17,200 x *g* for 2 min. Supernatant was removed. The pellet was reconstituted in 500 µL of ice-cold acetone and placed in a sonicating bath (FB15050, Elma Schmidbauer) for 30 sec. Samples were incubated at -20°C for 5 min before being centrifuged at 17,200 x *g* for 2 min. Acetone was carefully removed, before another complete acetone wash step was completed. 200 µL guanidine solution was added to each sample before sonication for 30 sec. Samples were centrifuged briefly to pellet any unsolubilised material, and 100 µL of supernatant was transferred to a 96-well plate. OD was measured at 375 nm in a FLUOstar Omega plate reader (BMG Labtech).

*RT-PCR*

RNA was extracted from 30 mg frozen cardiac and liver samples using the RNeasy fibrous tissue mini kit (Qiagen, Manchester, UK), as per manufacturer’s instructions. 300 μL buffer RLT, 1% β-mercaptoethanol, was added to previously weighed samples. Tissue was disrupted and homogenised in 300 μL buffer RLT, containing 10 μL β-mercaptoethanol per 1 mL buffer, using steel beads and a Tissue Lyser (Qiagen) for 2 min at 20 Hz. RNase free water (590 μL) was added to each sample followed by 10 μL proteinase K (around 6 mAU). Samples were then incubated at 55°C for 10 min before centrifugation at 10,000 x *g* for 3 min. Supernatant was taken forward and 0.5 volumes of 100% EtOH were added. Samples were transferred to RNeasy mini spin columns and centrifuged (8000 x *g* for 15 sec). The column membrane was washed with 350 μL Buffer RW1 before centrifuging, again as above. DNase I mastermix diluted in Buffer RDD was incubated directly onto the membrane for 15 min at 20-30°C. The membrane was then washed again in 350 μL Buffer RW1 before centrifugation.

Salts were removed from the membrane through two sequential washes with 500 μL Buffer RPE and centrifugation. Columns were then centrifuged at full speed for 1 min to remove all traces of buffers. RNA was eluted from the column using RNase free water, 80 μL total with centrifugation at 8000 x *g* for 1 min. RNA was then quantified using a Nanodrop 2000 spectrophotometer (Thermo Fisher Scientific). The RNA was determined to be of sufficient purity when a 260/280 value >1.8 and a 260/230 value around 2 were obtained.

The next step involved converting the RNA to cDNA using the QuantiTect Reverse Transcription kit (Qiagen), as per the manufacturer’s instructions. 1 μg RNA was added to a 96 well plate and the volume was made up to 12 μL using RNase free water. 2 μL gDNA wipeout per well were added and samples were incubated for 2 min at 42°C before immediately being placed on ice. 5 μL master mix containing 5x Quantinova RT buffer and RT primer mix (1:5) was added to each sample followed by 1 μL Quantiscript Reverse Transcriptase. The exception was a Non-Reverse-Transcriptase Control (NRTC) which did not include the Reverse Transcriptase. The plate was incubated for 15 min at 42°C followed by 3 min at 95°C to terminate the reaction by inactivating the Reverse Transcriptase. All incubations were performed using the QuantStudio 1 Real-Time PCR System (Applied Biosystems, Thermo Fisher Scientific).

Real time qPCR was conducted using the QuantiNova SYBR Green PCR kit (Qiagen) and QuantiTect Primer Assays (Qiagen). The details of specific primers are given in Supplementary Table 1. Each reaction well contained 5 μL QuantiNova SYBR Green Master Mix and 1 μL of the QuantiTect Primer Assay for the gene of interest. 1 μL of cDNA, that had previously been diluted 8-fold in diluted QuantiNova Yellow Template Dilution Buffer, was also added and the final reaction volume was made up to 10 μL with RNase free water (3 μL). In addition to the individual samples, 1 μL of each sample cDNA was pooled together. A serial dilution of this pooled cDNA was performed at 1:2 with RNase free water. Furthermore, a non-template control (no template cDNA) and a non-reverse transcriptase control (not exposed to reverse transcriptase during cDNA generation) were generated. Each individual sample, pooled sample serial dilution step, and control was plated in triplicate, with the corresponding SYBR Green Master Mix and primer for each gene, on a 384-well plate. qPCR was then carried out using a LightCyclerR 480 System (Roche), with an initial activation step at 95°C for 2 min followed by 40 cycles of 5 sec denaturation at 95°C and 10 sec annealing/extension step at 60°C.

A melt curve analysis was carried out using the LightCyclerR 480 Software (Roche). Genes of interest (*Vegfa, Ca9, Arg1, Higd1a*) were normalised to the geometric mean of three reference genes (*18S, Actb, Yhwaz*) and the fold change in gene expression was calculated using the relative standard curve method from the pooled samples.

*Statistics*

Results were analysed using a one-way analysis of variance (ANOVA) test to determine significant differences between experimental groups, unless otherwise stated. This was followed by a Tukey’s post-hoc test to compare individual group pairs. Where data was not normally distributed, e.g. analysis of amylin deposits by immunofluorescence, a Kruskal-Wallis test was used, followed by Dunn’s post-hoc test to compare individual group pairs. Differences were considered significant when p < 0.05.

**References**

1.	**Butler AE**, **Jang J**, **Gurlo T**, **Carty MD**, **Soeller WC**, **Butler PC**. Diabetes due to a progressive defect in beta-cell mass in rats transgenic for human islet amyloid polypeptide (HIP Rat): a new model for type 2 diabetes. *Diabetes* 53: 1509–1516, 2004. doi: 10.2337/diabetes.53.6.1509.

2.	**Cummings BP**, **Digitale EK**, **Stanhope KL**, **Graham JL**, **Baskin DG**, **Reed BJ**, **Sweet IR**, **Griffen SC**, **Havel PJ**. Development and characterization of a novel rat model of type 2 diabetes mellitus: the UC Davis type 2 diabetes mellitus UCD-T2DM rat. *Am J Physiol Regul Integr Comp Physiol* 295, 2008. doi: 10.1152/ajpregu.90635.2008.

3.	**Horscroft JA**, **Burgess SL**, **Hu Y**, **Murray AJ**. Altered oxygen utilisation in rat left ventricle and soleus after 14 days, but not 2 days, of environmental hypoxia. *PLoS One* 10: 1–16, 2015. doi: 10.1371/journal.pone.0138564.

4.	**Houle-Leroy P**, **Garland T**, **Swallow JG**, **Guderley H**. Effects of voluntary activity and genetic selection on muscle metabolic capacities in house mice Mus domesticus. *J Appl Physiol* 89: 1608–1616, 2000. doi: 10.1152/jappl.2000.89.4.1608.

5.	**Jha P**, **Wang X**, **Auwerx J**. Analysis of Mitochondrial Respiratory Chain Supercomplexes Using Blue Native Polyacrylamide Gel Electrophoresis (BN-PAGE). *Curr Protoc Mouse Biol* 6: 1–14, 2016. doi: 10.1002/9780470942390.mo150182.

6.	**Knapton AE**, **O’Brien KA**, **Sowton AP**, **Armstrong JL**, **Davies V**, **Kirk CM**, **McNally BD**, **Thackray BD**, **Holzner LM**, **Murray AJ**. Optimised measurement of mitochondrial respiratory capacity in frozen rat liver and heart. *Biochem Biophys Res Commun* 806, 2026. doi: 10.1016/j.bbrc.2026.153405.

7.	**Ly H**, **Verma N**, **Wu F**, **Liu M**, **Saatman KE**, **Nelson PT**, **Slevin JT**, **Goldstein LB**, **Biessels GJ**, **Despa F**. Brain microvascular injury and white matter disease provoked by diabetes-associated hyperamylinemia. *Ann Neurol* 82: 208–222, 2017. doi: 10.1002/ana.24992.

**Table 1:** Qiagen primers used

| **Target** | **NCBI Reference Sequence** | **GeneGlobe ID** |
| --- | --- | --- |
| *Actb* | NM_031144 | QT00193473 |
| *Arg1* | NM_017134 | QT00177611 |
| *Ca9* | NM_001107956 | QT01614522 |
| *Higd1a* | NM_080902 | QT00372428 |
| *Rn18s* | NR_046237 | QT02589300 |
| *Vegfa* | NM_001110335 | QT00198954 |
| *Ywhaz* | NM_013011 | QT01083992 |

**Supplementary Figure 1**

**A**	N-Linked	**B**	NS-Linked

**C**	S-Linked	**D**	Complex IV

*p = 0.06*

*

**Figure S1: Liver respiratory capacities expressed relative to citrate synthase activity. A)** N-linked respiration via complex I, supported by NADH; **B)** NS-linked respiration via complexes I&II, supported by NADH and succinate; **C)** S-linked respiration via complex II, supported by succinate; **D)** respiration via complex IV, supported by TMPD and ascorbate. Rates recorded as oxygen flux (pmol s^-1^ mg wet weight^-1^) and expressed relative to citrate synthase activity (mmol s^-1^ mg protein^-1^) from the same liver (n = 9-10 per group). Data represented as mean ± SEM. * p < 0.05

**Supplementary Figure 2**

**Figure S2: Cardiac electron transfer system (ETS) protein levels.** Cardiac levels of representative subunits of **A)** complex I (NDUFA9), **B)** complex II (SDHA), **C)** complex III (UQRC2), **D)** complex IV (MTCO1), **E)** ATP synthase (ATP5A), all expressed relative to WT (n = 5 per group) from wild-type (WT), hyperglycemic (UCD), human amylin-expressing (HIP) and amylin-knockout (AKO) rats. Data represented as mean ± SEM.

**Supplementary Figure 3**

**A**	N-Linked	**B**	NS-Linked

**C**	S-Linked	**D**	Complex IV

*

*

**Figure S3: Heart respiratory capacities expressed relative to citrate synthase activity. A)** N-linked respiration via complex I, supported by NADH; **B)** NS-linked respiration via complexes I&II, supported by NADH and succinate; **C)** S-linked respiration via complex II, supported by succinate; **D)** respiration via complex IV, supported by TMPD and ascorbate. Rates recorded as oxygen flux (pmol s^-1^ mg wet weight^-1^) and expressed relative to citrate synthase activity (mmol s^-1^ mg protein^-1^) from the same heart (n = 9-10 per group). Data represented as mean ± SEM. * p < 0.05
